# Medical Species Used in Russia for the Management of Diabetes and Related Disorders

**DOI:** 10.3389/fphar.2021.697411

**Published:** 2021-07-20

**Authors:** Alexander N. Shikov, Igor A. Narkevich, Alexandra V. Akamova, Oksana D. Nemyatykh, Elena V. Flisyuk, Vladimir G. Luzhanin, Mariia N. Povydysh, Iuliia V. Mikhailova, Olga N. Pozharitskaya

**Affiliations:** ^1^ Saint-Petersburg State Chemical Pharmaceutical University, Saint-Petersburg, Russia; ^2^ Perm State Pharmaceutical Academy, Perm, Russia; ^3^ Murmansk Marine Biological Institute of the Russian Academy of Sciences (MMBI RAS), Murmansk, Russia

**Keywords:** polyherbal mixture, herbal medicine, obesity, blood glucose, binary combination, triple combination, mechanisms of activity, synergy

## Abstract

**Background:** Polyherbal mixtures called “medical species” are part of traditional and officinal medicine in Russia. This review aimed to analyze medical species used in Russia for the treatment of diabetes and related disorders. The information relevant to medical species, diabetes, and obesity was collected from local libraries, the online service E-library.ru, and Google Scholar. The prediction of the antidiabetic activity for the principal compounds identified in plants was performed using the free web resource PASS Online.

**Results:** We collected and analyzed information about the compositions, specificities of use, and posology of 227 medical species. The medical species represent mixtures of 2–15 plants, while the most frequently mentioned in the literature are species comprising 3–6 plants. The top 10 plants among the 158 mentioned in the literature include *Vaccinium myrtillus* L., *Phaseolus vulgaris* L., *Taraxacum campylodes* G.E. Haglund., *Urtica dioica* L., *Rosa* spp., *Hypericum* spp*., Galega officinalis* L., *Mentha × piperita* L., *Arctium* spp, and *Fragaria vesca* L. The leading binary combination found in medical species comprises the leaves of *V. myrtillus* and pericarp of *P. vulgaris*; leaves of *V. myrtillus* and leaves of *U. dioica*; and leaves of *V. myrtillus* and aerial parts of *G. officinalis*. In triple combinations, in addition to the above-mentioned components, the roots of *T. campylodes* are often used. These combinations can be regarded as basic mixtures. Other plants are added to improve the efficacy, treat associated disorders, improve gastrointestinal function, prevent allergic reactions, etc. Meanwhile, an increase in plants in the mixture necessitates advanced techniques for quality control. A feature of medical species in Russia is the addition of fresh juices, birch sap, seaweeds, and adaptogenic plants. Modern studies of the mechanisms of action and predicted activities of the principal compounds from medicinal plants support the rationality of polyherbal mixtures. Nevertheless, the mechanisms are not well studied and reported due to the limited number of compounds. Further investigations with calculations of synergistic or additive indices are important for strengthening the scientific fundamentals for the wider use of medical species in the therapy of diabetes. Two medical species, “Arfazetin” (7 medicinal plants) and “Myrphasinum” (12 medicinal plants), are approved for use in officinal medicine. The efficacy of these species was confirmed in several *in vivo* experiments and clinical trials. According to modern regulatory rules, additional experiments and clinical trials are required for more detailed investigations of the mechanisms of action and confirmation of efficacy.

**Conclusion:** We believe that the scientifically based utilization of rich plant resources and knowledge of Russian herbal medicine can significantly contribute to the local economy as well as to the sectors seeking natural healing products.

## Introduction

Disorders of carbohydrate and lipid metabolism predispose individuals to diseases of the endocrine system, particularly diabetes. The rapid increase in patients with diabetes is one of the gravest and fastest-growing public health problems in the world. About 463 million people currently suffer from diabetes globally (IDF, 2019), of whom over 60 million were in European countries (Timmis et al., 2020), 34.2 million in the US (Centers for Disease Control and Prevention, 2020), and 4.5 million in Russia in 2017 (Dedov et al., 2018).

The mean cost of the development of a new drug from concept to market is considered to range from $314 million to $2.8 billion (Wouters et al., 2020). According to recent literature data, 50–70% of all the small-molecule therapeutics in clinical use today trace their origins to natural products (Newman and Cragg, 2020). Medicines derived from natural sources exhibit greater ranges of structural and physicochemical features that have been tailored through evolution for selective binding to functional macromolecules of the human body (Stratton et al., 2015). The intensive exploration of natural resources and utilization of the knowledge of traditional medicine provides an opportunity to reduce the time needed for development and keep costs reasonably low.

About four billion people around the world believe that, as “natural” products, herbal medicinal products (HMP) are “safe” or “safer” than conventional drugs and have turned to phytotherapeutics (Ekor, 2014). For centuries, Russia has been regarded a “herbophilious” society in which plants have been used as one of the primary foods and for the treatment of different diseases (Shikov et al., 2017). It is estimated that 58–60% of the population of Russia relies on HMP for the prophylaxis or treatment of different diseases (Shikov et al., 2011; Sammons et al., 2016). HMP currently makes up 20% of the Russian market for drugs (Akamova et al., 2017).

Medicinal plants have been effectively used for the treatment of diabetes in different systems of traditional as well as officinal medicine (Anzar, 2013; Shikov et al., 2014; Suzuki et al., 2017; Xiao and Luo, 2018; Okovitiy et al., 2018; Skalli et al., 2019; Salehi et al., 2019). The philosophy “one disease, one target, one drug” oversimplifies the mechanisms of disease and is becoming increasingly inefficient (Ulrich-Merzenich, 2014; Panossian et al., 2018; Shikov et al., 2018). Due to its multifactorial etiology, the holistic treatment of diabetes requires multi-pathway understanding and multi-targeting approaches. Modern network pharmacology studies emphasize the importance of the network-targeting, multicomponent therapy used in traditional Indian systems of medicine (Mukherjee et al., 2018; Banerjee et al., 2019), traditional Chinese medicine (Li et al., 2014; Xue et al., 2019), Kampo (Suzuki et al., 2017), etc.

Multicomponent or polyherbal mixtures for the treatment of diabetes are presented in different systems of Eastern traditional medicine (Namdul et al., 2001; Sato, 2004; Tong et al., 2012; Malgaonkar et al., 2016; Ghadge and Kuvalekar, 2017; Suzuki et al., 2017; Xu et al., 2019) as well as in European herbal medicine (Madić et al., 2021). Such traditional formulations include carefully selected leaves, stems, flowers, roots, seeds, sometimes minerals, and animal products. The main goal of complicated mixtures is to increase therapeutic efficacy and minimize toxicity.

Russian herbal medicine has adopted Eastern philosophy and a Western pragmatic approach. Herbal medicine in Russia is part of officinal medicine. According to the 14th edition of the State Pharmacopoeia of the Russian Federation, which became effective in 2018, an HMP has been defined as “a medicinal product manufactured or prepared from one kind of medicinal plant material or several kinds of raw materials and marketed in consumer-ready packaging form” (The State Pharmacopoeia of Russian Federation, 2018). The general monograph (OFS.1.4.1.0020.15) is devoted to polyherbal mixtures, which are defined with the specific term “medical species”. A “medical species” is a formulation representing a mixture of two or more types of integral, cut, or powdered medicinal plant materials, sometimes with the addition of mineral, synthetic, plant, or animal-derived substances. Medical species are used for the preparation of aqueous decoctions/infusions, occasionally in pure form as powders, powders for insufflation or ingestion, etc. (The State Pharmacopoeia of Russian Federation, 2018). Medical species have been known in Russia for centuries and were documented in the first herbalist manuscripts (Zmeev, 1896; Shikov et al., 2021). Apparently, due to their efficacy, the medical species were adopted from traditional medicine in officinal and were monographed in the first Russian military field “Pharmacopoeia Castrensis Rossica., 1765”. Thereafter, medical species were embodied in all the following pharmacopoeias of Russia.

The polyherbal mixtures used in Eastern systems of traditional medicine have gained global popularity, and several new medicinal products are being marketed in different countries. However, the potential of the medical species used in Russia remains little known and underestimated. This review aimed to analyze the medical species used in Russia for the treatment of diabetes and related disorders and enable a better understanding of the rationality of plant combinations.

Information on medical species was collected from the Pharmacopoeias of Russia (I–VI editions), Pharmacopoeias of USSR (VII–XI editions), and online State Register of Medicinal Preparations of the Russian Federation (2021). Guided by the keywords “traditional medicine + diabetes”, “phytotherapy + diabetes”, “traditional medicine + obesity”, and “phytotherapy + obesity”, we systematically searched the literature in library catalogs, on the online service E-library.ru, and on Google Scholar. Next, the publications were screened using the keyword combination “medical species”. Through this approach, 75 medical (herbalist) books were found. Some medical species were mentioned in different books. We provide several references for the same medical species in Table 1.

**TABLE 1:** The list of medical species used for the management of diabetes and related disorders in Russia.

Code^a^	Plant name, part used /(proportion)	Method of preparation	Recommended dosage	Indication	Reference
**2 plants**					
2.1	*Frangula alnus* Mill*.* bark; *Taraxacum campylodes* G.E.Haglund. roots; (10:3)	Decoction; 1 table spoon in 200 ml of boiling water	200 ml 2–3 times a day	Obesity	Safonov (2016)
2.2	*Vaccinium myrtillus* L. leaves; *Arctium* spp*.* ^b^ roots; (1:1)	Infusion; 10 g in 200 ml of boiling water	1 table spoon 3–4 times a day before eating	Diabetes	Sokolov and Zamotaiev (1984), Matkovskaya et al. (1988), Sinyakov (1992), Sinyakov (1999), Chirkov and Seryi (1993), Efimov and Shcherbak (1993), Tarasenko et al. (1998), Dontsov and Dontsov (2000), Sokolov (2000), Blinov (2000), Podduev (2001), Dremova et al. (2003), Nazina (2006), Davydovich et al. (2008), Bogdanova and Bashkirova (2010)
2.3	*Vaccinium myrtillus* L. leaves; *Phaseolus vulgaris* L. pericarp; (1:1)	Infusion; 1 table spoon in 200 ml of water, boil 15 min, maceration 30 min at room temp.	100 ml 3–4 times a day before eating	Diabetes	Sinyakov, (1992), Sinyakov, (1999), Efimov and Shcherbak (1993), Tarasenko et al. (1998), Podduev (2001), Smolianskii and Lifliandskii (2004), Korodetsky (2006), Davydovich et al. (2008), Balakirev (2010), Bogdanova and Bashkirova (2010)
2.4	*Inula helenium* L*.* roots; *Arctium lappa* L*.* roots; (1:1)	Infusion; 1 table spoon in 400 ml of water, boil 10 min	1 table spoon 3 times a day	Diabetes	Volynchenko (2003)
2.5	*Galega officinalis* L. aerial part; *Galega officinalis* L. seeds; (7:3)	Infusion; 1 tea spoon in 200 ml of water, boil 10 min, maceration 20–30 min,	200 ml 3 times a day 30 min before eating	Diabetes	Sinyakov (1999)
2.6	*Taraxacum campylodes* G.E.Haglund. roots; *Taraxacum campylodes* G.E.Haglund. leaves; (1:1)	Decoction; 6–10 g in 200 ml of water, boil 10 min, maceration 30 min	1 table spoon 3 times a day 30 min before eating	Diabetes	Podduev (2001)
2.7	*Taraxacum campylodes* G.E.Haglund. roots; *Mentha × piperita* L*.* leaves; (1:3)	Decoction; 4 tea spoons in 200 ml of water, boil 5–7 min, maceration 30 min	100 ml 3–4 times a day before eating	Diabetes
**3 plants**					
3.1	*Urtica dioica* L. leaves; *Juniperus communis* L. fruits; *Equisetum arvense* L. aerial part; (2:3:4)	Infusion; 1 table spoon in 500 ml of boiling water	66 ml 3 times a day before eating	Obesity	Osetrov and Shreter (2001)
3.2	*Ononis spinosa* L. roots; *Taraxacum campylodes* G.E.Haglund. roots; *Frangula alnus* Mill*.* bark; (3:3:10)	Infusion; 3 table spoons in 600 ml of boiling water	200 ml 2–3 times a day before eating	Obesity	Lager (1991), Lager (2002), Efimov and Shcherbak (1993), Kukes (1999), Dontsov and Dontsov (2000), Bubenchikova et al. (2003)
3.3	*Tussilago farfara* L. leaves; *Betula* spp*.* ^c^ leaves; *Rubus* *caesius* L. leaves; 1:1:8	Infusion; 10 g in 200 ml of boiling water	200 ml 2 times a day before eating	Obesity	Dontsov and Dontsov (2000), Sokolov (2000), Kiyanova (2005), Maznev (2005)
3.4	*Phaeophyceae* (*Cystoseira barbata* (Stackh.) C.Agardh) thallus; *Pimpinella anisum* L. fruits; *Glycyrrhiza glabra* L*.* roots; (2:1:1)	Decoction; 2 table spoons in 500 ml of water	100 ml 3–4 times a day	Obesity	Yordanov et al. (1972), Chirkov and Seryi (1993), Kiyanova (2005), Maznev (2005)
3.5	*Achillea millefolium* L. aerial part; *Hypericum perforatum* L. aerial part; *Phaeophyceae* (*Cystoseira barbata* (Stackh.) C.Agardh) thallus; (2:2:1)	Infusion (herbal tea); 2 table spoons in 400 ml boiling water	100 ml 3–4 times a day	Obesity	Yordanov et al. (1972), Chirkov and Seryi (1993), Efimov and Shcherbak (1993)
3.6	*Frangula alnus* Mill. bark; *Achillea millefolium* L*.* aerial part; *Juniperus communis* L. fruits; (3:2:1)	Infusion; 2 table spoons in 500 ml of boiling water	200 ml 3 times a day	Obesity	Lager (1991), Lager (2002), Dontsov and Dontsov (2000), Rendiuk (2006), Safonov (2016)
3.7	*Vaccinium myrtillus* L. leaves; *Urtica dioica* L. leaves; *Sambucus nigra* L. leaves; (2:1:1)	Decoction; 1 table spoon in 200 ml boiling water	150 ml a day	Lowering of blood glucose level	Sokolov and Zamotaiev (1984), Matkovskaya et al. (1988), Sinyakov (1992), Sinyakov (1999); Efimov and Shcherbak (1993), Tarasenko et al. (1998), Kukes (1999), Blinov (2000), Dontsov and Dontsov (2000), Sokolov (2000), Bubenchikova et al. (2003), Podduev (2001), Onipko (2002), Davydovich et al. (2008), Bogdanova and Bashkirova (2010)
3.8	*Equisetum arvense* L*.* aerial part; *Polygonum aviculare* L*.* aerial part; *Fragaria vesca* L*.* leaves; (1:2:1)	Infusion; 1 table spoon in 400 ml hot water	400 ml a day	Lowering of blood glucose level	Sokolov and Zamotaiev (1984), Matkovskaya et al. (1988), Sinyakov (1992), Sinyakov (1999); Chirkov and Seryi (1993), Nikultseva (1994), Tarasenko et al. (1998), Trofimenko and Mogilny (1998), Kukes (1999), Dontsov and Dontsov (2000), Sokolov (2000), Bubenchikova et al. (2003), Turishchev (2000), Blinov (2000), Podduev (2001), Dremova et al. (2003), Turishchev (2005), Nazina (2006), Davydovich et al. (2008), Volynchenko (2003), Ruzhenkova (2014), Maznev (2014)
3.9	*Vaccinium myrtillus* L. leaves; *Urtica dioica* L. leaves; *Taraxacum campylodes* G.E.Haglund. roots; (1:1:1)	Infusion (herbal tea); 10 g in 200 ml of boiling water	100 ml 3 times a day before eating	Lowering of blood glucose level	Sokolov and Zamotaiev (1984), Matkovskaya et al. (1988), Sinyakov (1992), Chirkov and Seryi (1993), Efimov and Shcherbak (1993), Nikolaychuk (1997), Fedyukovich (1998), Kukes (1999), Bubenchikova et al. (2003), Turishchev (2000), Sokolov (2000), Blinov (2000), Dontsov and Dontsov (2000), Podduev (2001), Onipko (2002), Nikolaychuk and Zubitskaya (2003), Dremova et al. (2003), Brusenskaya and Kaz’min (2005), Turishchev (2005), Nazina (2006), Davydovich et al. (2008), Bogdanova and Bashkirova (2010), Pigulevskaya (2018)
3.10	*Hypericum perforatum* L. flowers; *Galega officinalis* L. aerial part; *Urtica dioica* L. leaves; (5:4:3)	Infusion; 60 g in 200 ml of boiling water	100 ml or 66 ml 4 times a day before eating	Diabetes	Brusenskaya and Kaz’min (2005)
3.11	*Hypericum perforatum* L*.* aerial part; *Mentha × piperita* L*.* leaves; *Vaccinium myrtillus* L. leaves; (1:1:1)	Infusion; 1 table spoon in 250 ml of boiling water	125 ml 2 times a day before eating	Diabetes	Osetrov and Shreter (2001)
3.12	*Vaccinium myrtillus* L. leaves; *Elymus repens*(L.)rhizomes; *Rubus* *caesius* L. roots; (250:10:2)	Infusion; 262 g in 1,500 ml of boiling water	During the day instead of water	Diabetes	Osetrov (1993), Osetrov and Shreter (2001)
3.13	*Vaccinium vitis-idaea* L. leaves; *Ruta graveolens* L. leaves; *Angelica archangelica* L. roots; (5:3:2)	Infusion; 1 table spoon in 200 ml of boiling water, boil 10 min, maceration 30–40 min at room temp.	100 ml 3–4 times a day 30 min before eating	Diabetes	Sinyakov (1992), Sinyakov (1999), Efimov and Shcherbak (1993), Tarasenko et al. (1998), Bogdanova and Bashkirova (2010)
3.14	*Arctostaphylos uva-ursi* (L.) Spreng. leaves; *Valeriana officinalis* L. roots and rhizomes*; Vaccinium myrtillus* L. leaves; (1:1:2)	Infusion; 1 table spoon in 200 ml of boiling water, boil 15 min, maceration 30 min at room temp.	200 ml 3–4 times a day before eating	Diabetes	Tarasenko et al. (1998), Podduev (2001)
3.15	*Fragaria vesca* L. leaves; *Cichorium intybus* L*.* leaves; *Sambucus nigra* L. flowers; (2:2:1)	Infusion; 1 table spoon in 200 ml of boiling water, boil 5 min, maceration 1 h at room tempature	66 ml 3 times a day 20 min before eating	Diabetes	Efimov and Shcherbak (1993), Nikolaychuk (1997), Rendiuk (2006), Bogdanova and Bashkirova (2010)
3.16	*Vaccinium myrtillus* L. leaves; *Fragaria vesca* L. leaves; *Rubus* *caesius* L. leaves; (1:1:1)	Infusion; 1 table spoon in 300 ml of boiling water, boil 3 min, maceration 10 min at room tempature	100 ml 3 times a day 20 min before eating	Diabetes	Efimov and Shcherbak (1993), Nikolaychuk (1997), Fedyukovich (1998), Nikolaychuk and Zubitskaya (2003), Dremova et al. (2003), Rendiuk (2006), Bogdanova and Bashkirova (2010), Pigulevskaya (2018)
3.17	*Vaccinium myrtillus* L. leaves; *Phaseolus vulgaris* L. pericarp; *Matricaria chamomilla* L. flowers; (1:2:1)	Infusion; 40 g in 400 ml of boiling water, maceration 5–6 h at room tempature	100 ml 4 times a day 20–30 min before eating	Diabetes	Lavrenova and Lavrenov (2007)
3.18	*Arctium* spp.^b^ roots; *Phaseolus vulgaris* L. pericarp; *Vaccinium myrtillus* L. leaves; (1:1:1)	Infusion; 60 g in 1,000 ml of cold water, maceration 12 h at room temperature, boil 5 min, maceration 1 h	150 ml 5 times a day 1 h after eating	Diabetes	Seredin and Sokolov (1973), Lager (1991), Lager (2002), Makhlayuk (1991), Chirkov and Seryi (1993), Dmitriev et al. (1994) Nikultseva (1994), Fedyukovich (1998), Podduev (2001), Pirogov (2008), Grechanyi (2013), Melik-Gusseinov and Rekkandt (2014), Maznev (2014)
3.19	*Vaccinium myrtillus* L. leaves; *Galega officinalis* L. aerial part; *Urtica dioica* L. leaves; (1:1:1)	Infusion; 1 table spoon in 300 ml of boiling water	2 table spoons 3–4 times a day 20 min before eating	Diabetes	Sinyakov (1992), Sinyakov (1999), Efimov and Shcherbak (1993), Nikultseva (1994), Nikolaychuk (1997), Trofimenko and Mogilny (1998), Fedyukovich (1998), Blinov (2000), Onipko (2002), Nikolaychuk and Zubitskaya (2003), Nazina (2006), Bogdanova and Bashkirova (2010), Maznev (2014)
3.20	*Vaccinium myrtillus* L. leaves; *Taraxacum campylodes* G.E.Haglund. leaves; *Galega officinalis* L. aerial part; (1:1:1)	Infusion; 1 table spoon in 300 ml of boiling water	100 ml 2–3 times a day 20 min before eating	Diabetes	Sinyakov (1992), Sinyakov (1999), Trofimenko and Mogilny (1998), Blinov (2000), Nazina (2006)
3.21	*Leonurus* spp*.* ^d^leaves; *Fragaria vesca* L. leaves; *Morus alba* L. leaves; (1:2:4)	Infusion; 1 table spoon in 200 ml of boiling water	2 table spoons 3 times a day after eating	Diabetes	Sinyakov (1992), Sinyakov (1999), Nikultseva (1994), Nikolaychuk (1997), Tarasenko et al. (1998), Trofimenko and Mogilny (1998), Fedyukovich (1998), Blinov (2000), Podduev (2001), Dremova et al. (2003), Kiyanova (2005), Nazina (2006), Davydovich et al. (2008), Bogdanova and Bashkirova (2010), Maznev (2014)
3.22	*Vaccinium myrtillus* L. leaves; *Inula helenium* L. roots; *Polygonum aviculare* L. aerial part; (1:1:1)	Decoction; 1 table spoon in 200 ml of water	50 ml 2–3 times a day	Diabetes	Chirkov and Seryi (1993), Kukes (1999), Bubenchikova et al. (2003), Dremova et al. (2003)
3.23	*Vaccinium myrtillus* L. leaves; *Taraxacum campylodes* G.E.Haglund. leaves; *Artemisia vulgaris*L., aerial part; (5:5:4)	Decoction; 1 table spoon in 300 ml of water, boil 5 min, maceration 30 min	100 ml 3–4 times a day	Diabetes	Podduev (2001)
3.24	*Vaccinium myrtillus* L. leaves; *Phaseolus vulgaris* L. pericarp; *Mentha × piperita* L. leaves; (1:1:1)	Infusion; 2 table spoons in 500 ml of boiling water, maceration 30 min	70 ml 3 times a day before eating	Diabetes	Podduev (2001)
3.25	*Avena sativa* L. aerial part in flowering phase; *Vaccinium myrtillus* L. leaves; *Phaseolus vulgaris* L*.* pericarp; (1:2:2)	Infusion; 1 table spoon in 200 ml of boiling water	200 ml 3–4 times a day before eating	Diabetes accompanied by impotence in men	Sklyarevsky and Gubanov (1989), Efimov and Shcherbak (1993), Blinov (2000); Orlova, (2001), Dremova et al. (2003), Brusenskaya and Kaz’min (2005), Kiyanova (2005), Rendiuk (2006), Davydovich et al. (2008), Bogdanova and Bashkirova (2010)
3.26	*Helichrysum arenarium* (L.) Moench. flowers; *Fagopyrum esculentum* Moench flowers and leaves; *Vaccinium myrtillus* L. leaves; (1:1:2)	Infusion; 12 g in 1,000 ml of boiling water, maceration 5–6 h at room temp., then 15 min in boil water bath	200 ml (warm) with 10 g of honey 3–4 times a day before eating	Diabetes accompanied by impotence in men	Brusenskaya and Kaz’min (2005)
3.27	*Urtica dioica* L. leaves; *Inula helenium* L*.* roots; Sugar; (9:1:5)	Decoction; 2 table spoons in 200 ml of water	100 ml (warm ) 3 times a day before eating	Metabolism improving	Chirkov and Seryi (1993)
3.28	*Viola tricolor* L. aerial part; *Bidens tripartite* L. aerial part; *Solanum dulcamara* L. aerial part; (4:4:1)	Infusion (herbal tea); 1 table spoon in 200 ml of boiling water	4 table spoon 3–4 times a day	King’s evil, metabolic disorder	Chirkov and Seryi (1993)
**4 plants**					
4.1	*Mentha × piperita* L. leaves; *Foeniculum vulgare* Mill. fruits; *Matricaria chamomilla* L. flowers; *Tilia cordata* Mill. flowers; (4:3:3:3)	Infusion; 10 g in 200 ml of boiling water	200 ml 2–3 times a day	Obesity	Safonov (2016)
4.2	*Levisticum officinale* W.D.J.Koch roots; *Juniperus communis* L. fruits; *Phaeophyceae* (*Cystoseira barbata* (Stackh.) C.Agardh) thallus; *Achillea millefolium* L. aerial part; (1:1:1:1)	Decoction; 2 table spoons in 500 ml of water	132 ml or 200 ml 2–3 times a day	Obesity	Chirkov and Seryi (1993)
4.3	*Ononis spinosa* L. roots; *Persicaria hydropiper* (L.) Delarbre aerial part; *Foeniculum vulgare* Mill. fruits; *Alchemilla xanthochlora* Rothm. roots and aerial part; (6:1:1:1)	Decoction; 2 table spoons in 500 ml of water	100 ml 4 times a day before eating	Obesity	Chirkov and Seryi (1993)
4.4	*Artemisia absinthium* L*.,* aerial part; *Salvia officinalis* L. leaves; *Rosmarinus officinalis*L.leaves;*Prunus spinosa*L. flowers; (1:1:1:1)	Infusion; 3 table spoons in 500 ml of boiling water	150 ml 3 times a day	Obesity	Osetrov and Shreter (2001)
4.5	*Frangula alnus* Mill. bark; *Taraxacum campylodes* G.E.Haglund.roots; *Petroselinum crispum* (Mill.) Fuss fruits; *Foeniculum vulgare* Mill. fruits; (3:1:1:1)	Infusion; 20 g in 400 ml of boiling water	400 ml in the morning before eating	Obesity	Dontsov and Dontsov (2000), Maznev (2005), Kiyanova (2005)
4.6	*Apium graveolens* L. leaves*; Phaseolus vulgaris* L. pericarp; *Humulus lupulus* L. fruits; *Pastinaca sativa* L. root; (4:4:3:1)	Herbal tea; 1 table spoon in 200 ml of boiling water	30 ml 6 times a day	Obesity and diabetes	Protasenya and Vasilenko (1992)
4.7	*Nasturtium officinale*R.Br.aerial part;*Morus nigra*L.leaves;*Urtica dioica* L. leaves; *Phaseolus vulgaris* L. pericarp; (1:1:1:1)	Infusion; 1 table spoon in 300 ml of boiling water	100 ml 3 times a day before eating	Diabetes	Osetrov (1993)
4.8	*Phaseolus vulgaris* L. pericarp; *Betula* spр.d^d^ leaves; *Taraxacum campylodes* G.E.Haglund. roots; *Sinapis alba*L. seeds; (8:12:4:1)	Infusion; 1 table spoon in 300 ml of boiling water	150 ml 2 times a day	Diabetes	Osetrov (1993), Maznev (2014)
4.9	*Vaccinium myrtillus* L. leaves; *Rubus caesius* L. leaves; *Fragaria vesca* L. leaves; *Rosa majalis* Herrm. fruits; (1:1:1:1)	Infusion; 1 table spoon in 200 ml of boiling water, maceration 30 min	100 ml 3 times a day before eating	Diabetes	Efimov and Shcherbak (1993), Nikolaychuk (1997), Fedyukovich (1998), Onipko (2002), Nikolaychuk and Zubitskaya (2003), Smolianskii and Lifliandskii (2004), Bogdanova and Bashkirova (2010)
4.10	*Juniperus communis* L. fruits; *Linum usitatissimum* L. seeds; *Vaccinium myrtillus* L. leaves; *Vaccinium vitis-idaea* L. leaves; (1:1:1:1)	Infusion; 1 tea spoon in 200 ml of boiling water, boil 5 min, maceration 30 min	50 ml 3 times a day before eating	Diabetes	Nikolaychuk and Zubitskaya (2003), Smolianskii and Lifliandskii (2004)
4.11	*Galega officinalis* L. aerial part; *Urtica dioica* L. leaves; *Taraxacum campylodes* G.E.Haglund. roots; *Phaseolus vulgaris* L. pericarp; (1:1:1:1)	Infusion; 1 table spoon in 200 ml of boiling water	50 ml 3 times a day 15 min before eating	Diabetes	Dontsov and Dontsov (2000), Podduev (2001), Vinogradova et al. (2001)
4.12	*Vaccinium myrtillus* L. leaves; *Fragaria vesca* L. leaves; *Tilia cordata* Mill. flowers; *Verbascum densiflorum* Bertol. flowers; (8:5:4:3)	Infusion; 2 table spoons in 400 ml of boiling water	100–132 ml 3 times a day 30 min before eating	Diabetes	Sinyakov (1992), Efimov and Shcherbak, (1993), Tarasenko et al. (1998), Podduev (2001), Dremova et al. (2003), Bogdanova and Bashkirova (2010), Volynchenko (2003)
4.13	*Vaccinium myrtillus* L. leaves; *Urtica dioica* L. leaves; *Taraxacum campylodes* G.E.Haglund. roots; *Phaseolus vulgaris* L. pericarp; (1:1:1:1)	Infusion; 2 table spoons in 500 ml of boiling water, boil 12–15 min, maceration 30–40	66 ml 3 times a day 30 min before eating	Diabetes	Sinyakov (1992), Efimov and Shcherbak (1993), Dmitriev et al. (1994), Tarasenko et al. (1998), Kukes (1999), Sinyakov (1999), Bubenchikova et al. (2003), Bogdanova and Bashkirova (2010), Melik-Gusseinov and Rekkandt (2014)
4.14	*Equisetum arvense* L*.* aerial part; *Polygonum aviculare* L*.* aerial part; *Urtica dioica* L*.* leaves; *Capsella bursa-pastoris* (L.) Medik. aerial part; (1:1:1:1)	Infusion; 2 table spoons in 500 ml of boiling water, boil 3–5 min, maceration 30–40 min	40–50 ml 3–4 times a day 30 min before eating	Diabetes	Sinyakov (1992), Sinyakov (1999); Nikolaychuk (1997), Tarasenko et al. (1998), Podduev (2001), Nikolaychuk and Zubitskaya (2003), Davydovich et al. (2008), Maznev (2014), Pigulevskaya (2018)
4.15	*Polygonum aviculare* L*.* aerial part; *Equisetum arvense* L*.* aerial part; *Fragaria vesca* L*.* leaves; *Aralia elata* (Miq.) Seem roots; (7:5:5:2)	Infusion; 2 table spoons in 500 ml of boiling water, boil 3–5 min, maceration 20–30 min	40–50 ml 3–4 times a day 30 min before eating	Diabetes	Sinyakov (1992), Tarasenko et al. (1998), Podduev (2001)
4.16	*Taraxacum campylodes* G.E.Haglund. roots; *Phaseolus vulgaris* L. pericarp; *Hypericum perforatum* L*.* aerial part; *Vaccinium myrtillus* L. leaves; (1:1:1:1)	Infusion; 2 table spoons in 500 ml of boiling water, maceration 12 h in thermos	100 ml 3 times a day 30 min before eating	Diabetes	Sinyakov (1992), Sinyakov (1999); Tarasenko et al. (1998)
4.17	*Vaccinium myrtillus* L. leaves; *Phaseolus vulgaris* L. pericarp; *Arctium lappa* L. roots; *Vaccinium vitis-idaea* L. leaves; (2:2:1:1)	Infusion; 1 table spoon in 200 ml of boiling water, boil 15 min, maceration 30 min	200 ml 3–4 times a day before eating	Diabetes	Tarasenko et al. (1998), Podduev (2001)
4.18	*Rubus* *caesius* L. leaves;*Vaccinium vitis-idaea* L. leaves; *Primula veris* L. leaves; *Galega officinalis* L. aerial part; (3:3:2:4)	Infusion; 1 table spoon in 300 ml of boiling water, boil 3 min, maceration at room tempature	100 ml 3 times a day after eating	Diabetes	Efimov and Shcherbak (1993), Nikolaychuk (1997), Fedyukovich (1998), Dremova et al. (2003), Rendiuk (2006)
4.19	*Centaurium erythraea* Rafn aerial part; *Vaccinium myrtillus* L. leaves; *Equisetum arvense* L*.* aerial part; *Polygonum aviculare* L. aerial part; (1:1:1:1)	Infusion; 1 table spoon in 200 ml of boiling water, boil 5 min, maceration at room tempature	200 ml 2–3 times a day before eating	Diabetes	Efimov and Shcherbak (1993), Nikolaychuk (1997), Fedyukovich (1998), Onipko (2002), Nikolaychuk and Zubitskaya (2003), Rendiuk (2006), Bogdanova and Bashkirova (2010)
4.20	*Arctium lappa* L. roots; *Cichorium intybus* L. roots; *Valeriana officinalis* L. roots and rhizomes; *Rubus caesius* L. root; (2:3:3:1)	Herbal tea; 3 table spoons in 1,000 ml of boiling water	100 ml 7 times a day	Diabetes	Rendiuk (2006)
4.21	*Vaccinium myrtillus* L. leaves; *Phaseolus vulgaris* L. pericarp; *Arctium lappa* L. roots; *Vaccinium vitis-idaea* L. leaves; (2:2:1:1)	Infusion; 1 table spoon in 200 ml of boiling water, boil 15 min, maceration 30 min	200 ml 3–4 times a day before eating	Diabetes	Tarasenko et al. (1998)
4.22	*Cichorium intybus* L. roots; *Plantago major* L. leaves; *Arctium lappa* L. roots; *Equisetum arvense* L. aerial part; (1:1:1:1)	Infusion; 1 table spoon in 300 ml of boiling water, boil 3 min, maceration 10 min	66 ml 3 times a day 20 min before eating	Diabetes	Efimov and Shcherbak (1993), Nikolaychuk (1997), Fedyukovich (1998), Onipko (2002), Nikolaychuk and Zubitskaya (2003), Rendiuk (2006), Bogdanova and Bashkirova (2010), Pigulevskaya (2018)
4.23	*Phaseolus vulgaris* L. pericarp; *Vaccinium myrtillus* L. leaves; *Taraxacum campylodes* G.E.Haglund. roots; *Urtica dioica* L. leaves; (1:1:1:1)	Infusion; 1 table spoon in 200 ml of boiling water, maceration 20 min	200 ml 3–4 times a day	Diabetes	Volynchenko (2003)
4.24	*Juglans regia* L. leaves; *Vaccinium myrtillus* L. leaves; *Phaseolus vulgaris* L. pericarp; *Arctium* spp.^b^ roots; (1:1:1:1)	Infusion; 1 table spoon in 200 ml of cold water, maceration 1–2 h at room tempature, boil 5–7 min	200 ml 5–6 times a day after eating	Diabetes	Volynchenko (2003)
4.25	*Vaccinium myrtillus* L. fruits; *Sambucus nigra* L. flowers; *Arctium lappa* L*.* roots; *Zea mays* L. corn silk; (1:1:1:1)	Decoction	1–2 table spoons 3 times a day 30 min before eating for 1–1.5 months	Diabetes	Kukes (1999)
4.26	*Vaccinium myrtillus* L. leaves; *Phaseolus vulgaris* L. pericarp; *Polygonum aviculare* L*.* aerial part; *Arctostaphylos uva-ursi* (L.) Spreng. leaves; (1:1:1:1)	Infusion; 60 g in 300 ml of boiling water	66 ml 3 times a day	Diabetes	Lager (1991), Lager (2002), Efimov and Shcherbak (1993), Nikolaychuk (1997), Fedyukovich (1998), Nikolaychuk and Zubitskaya (2003), Bogdanova and Bashkirova (2010)
4.27	*Galega officinalis* L. aerial part; *Vaccinium myrtillus* L. leaves; *Urtica dioica* L. leaves; *Taraxacum campylodes* G.E.Haglund. roots; (1:1:1:1)	Infusion; 1 table spoon in 200 ml of boiling water	200 ml 3–4 times a day	Diabetes	Lager (1991), Lager, (2002), Efimov and Shcherbak (1993), Bogdanova and Bashkirova (2010)
4.28	*Phaseolus vulgaris* L. pericarp; *Galega officinalis* L. aerial part; *Betula pendula* Roth*.* leaves; *Vaccinium myrtillus* L. leaves;(1:1:1:1)	Infusion; 2 table spoons in 400 ml of boiling water, boil 10 min, maceration 30–40 min	100 ml 3 times a day 30 min before eating	Diabetes	Sinyakov (1999)
4.29	*Galega officinalis* L. aerial part; *Vaccinium vitis-idaea* L*.* leaves; *Frangula alnus* Mill. bark; *Betula pendula* Roth*.* Leaves; (40:40:10:10)	Infusion; 3 table spoons in 600 ml of boiling water, 15 min in boil water bath, maceration 30–40 min	130 ml 3 times a day	Diabetes	Sinyakov (1999)
4.30	*Galega officinalis* L. aerial part; *Vaccinium myrtillus* L. leaves; *Sambucus nigra* L. leaves; *Viscum album* L. aerial part; (7:7:4:2)	Infusion; 2 table spoons in 400 ml of boiling water, 15 min in boil water bath, maceration 30–40 min	50–130 ml 2–3 times a 30 min before eating	Diabetes	Sinyakov (1999)
4.31	*Vaccinium myrtillus* L. leaves; *Fragaria vesca* L. leaves; *Tilia cordata* Mill. flowers; *Verbascum densiflorum* Bertol. flowers; (8:5:4:3)	Infusion; 2 table spoons in 400 ml of boiling water, 15 min in boil water bath, maceration 30–40 min	50–130 ml 2–3 times a day 30 min before eating	Diabetes	Sinyakov (1999)
4.32	*Phaseolus vulgaris* L. pericarp; *Vaccinium myrtillus* L. leaves; *Laurus nobilis* L. leaves; *Morus alba* L. leaves; (1:1:1:1)	Infusion; 2–3 table spoons in 500 ml of boiling water, 15 min in boil water bath, maceration 30–40 min	200 ml 3–4 times a day 30 min before eating	Diabetes	Sinyakov (1999)
4.33	*Vaccinium vitis-idaea* L. leaves; *Taraxacum campylodes* G.E.Haglund. leaves; *Urtica dioica* L. leaves; *Galega officinalis* L. aerial part; (1:1:1:1)	Infusion; 2 table spoons in 500 ml of boiling water, boil 5–6 min, maceration 1–2 h	100 ml 2–3 times a day 20 min before eating	Diabetes	Sinyakov (1999)
4.34	*Vaccinium myrtillus* L. leaves; *Betula pendula* Roth*.* leaves; *Phaseolus vulgaris* L*.* pericarp; *Urtica dioica* L. leaves; (60:20:10:10)	Infusion; 2 table spoons in 500 ml of boiling water, boil 10 min, maceration 1–2 h	100 ml 3 times a day 20–30 min before eating	Diabetes	Sinyakov (1999)
4.35	*Phaseolus vulgaris* L. pericarp; *Vaccinium myrtillus* L. leaves; *Rosa* spp*.* ^6^ fruits; *Equisetum arvense* L. aerial part; (4:4:4:1)	Infusion; 65 g in 1,000 ml of water, boil 2 min, maceration 8–12 h in a dark place	100 ml 3 times a day before eating	Diabetes	Brusenskaya and Kaz’min (2005)
4.36	*Vaccinium myrtillus* L. leaves; *Hypericum* spp*.* ^e^aerial part; *Phaseolus vulgaris* L. pericarp; *Galega officinalis* L. aerial part; (4:4:4:5)	Infusion (herbal tea); 1 table spoon in 200 ml of boiling water	100 ml 2 times a day before eating	Diabetes	Efimov and Shcherbak (1993), Brusenskaya and Kaz’min (2005), Davydovich et al. (2008), Bogdanova and Bashkirova (2010)
4.37	*Avena sativa* L. aerial part; *Linum (usitatissimum* L.*)* seeds; *Phaseolus vulgaris* L. pericarp; *Vaccinium myrtillus* L. leaves; (1:1:1:1)	Infusion; 3 table spoons in 600 ml of boiling water	50 ml 6–8 times a day	Diabetes	Makhlayuk (1991), Sinyakov (1992), Chirkov and Seryi (1993), Dmitriev et al. (1994), Nikultseva (1994), Tarasenko et al. (1998), Trofimenko and Mogilny, (1998), Sinyakov (1999), Podduev (2001), Volynchenko (2003), Popov (2004), Lavrenova and Lavrenov (2007), Davydovich et al. (2008), Pirogov (2008), Grechanyi (2013), Maznev (2014), Melik-Gusseinov and Rekkandt (2014)
4.38	*Arctostaphylos uva-ursi* (L.) Spreng. leaves; *Galega officinalis* L. aerial part; *Vaccinium myrtillus* L. leaves; *Valeriana officinalis* L. roots; (1:1:1:1)	Infusion;1 tea spoon in 200 ml of boiling water	200 ml 3–4 times a day before eating	Diabetes	Yordanov et al. (1972), Matkovskaya et al. (1988), Lager (1991), Sinyakov (1992), Sinyakov (1999), Chirkov and Seryi (1993), Nikolaychuk (1997), Tarasenko et al. (1998), Nikolaychuk and Zubitskaya (2003), Kiyanova (2005), Davydovich et al. (2008), Maznev (2014)
4.39	*Betula pendula* Roth. leaves; *Frangula alnus* Mill*.* bark; *Vaccinium myrtillus* L. leaves; *Galega officinalis* L. aerial part; (1:1:4:4)	Infusion; 1 tea spoon in 200 ml of boiling water	200 ml 3–4 times a day before eating	Diabetes	Yordanov et al. (1972), Lager (1991), Larger (2002), Chirkov and Seryi (1993), Efimov and Shcherbak (1993), Dontsov and Dontsov (2000), Bogdanova and Bashkirova (2010)
4.40	*Vaccinium myrtillus* L. leaves; *Galega officinalis* L. aerial part; *Phaseolus vulgaris* L. pericarp; *Mentha × piperita* L*.* leaves; (1:1:1:1)	Infusion; 2 table spoons in 500 ml of boiling water	50–66 ml 3–4 times a day 30 min before eating	Diabetes	Yordanov et al. (1972), Matkovskaya et al. (1988), Lager (1991), Lager (2002); Sinyakov (1992), Sinyakov (1999), Chirkov and Seryi (1993), Efimov and Shcherbak (1993), Nikultseva (1994), Trofimenko and Mogilny (1998), Dontsov and Dontsov (2000), Blinov (2000), Dremova et al. (2003), Maznev (2005); Nazina (2006), Davydovich et al. (2008), Maznev (2014), Pigulevskaya (2018)
4.41	*Plantago major* L. leaves; *Taraxacum campylodes* G.E.Haglund. leaves; *Urtica dioica* L*.* leaves; *Vaccinium myrtillus* L. leaves; (1:1:1:1)	Infusion; 1 table spoon in 200 ml of boiling water	100 ml 3–4 times a day 20 min before eating	Diabetes	Sinyakov (1992), Sinyakov (1999), Efimov and Shcherbak (1993), Nikolaychuk (1997), Tarasenko et al. (1998), Fedyukovich (1998), Kukes (1999), Blinov (2000), Podduev (2001), Nikolaychuk and Zubitskaya (2003), Nazina (2006), Ryzhenko (2007), Davydovich et al. (2008), Bogdanova and Bashkirova (2010), Maznev (2014)
4.42	*Capsella bursa-pastoris* (L.) Medik. aerial part; *Equisetum arvense* L*.* aerial part; *Polygonum aviculare* L*.* aerial part; *Valeriana officinalis* L. roots and rhizomes; (1:1:1:1)	Infusion; 1 table spoon in 200 ml of boiling water	1 table spoon 3–4 times a day 20–30 min before eating	Diabetes	Blinov (2000), Nazina (2006)
4.43	*Galega officinalis* L. aerial part; *Juglans regia* L*.* leaves; *Mentha × piperita* L*.* leaves; *Polygonum aviculare* L. aerial part ; (1:1:1:1)	Infusion; 1 table spoon in 200 ml of boiling water	66 ml 3 times a day 15–20 min before eating	Diabetes	Sinyakov (1992), Sinyakov (1999), Efimov and Shcherbak (1993), Nikolaychuk (1997), Tarasenko et al. (1998), Fedyukovich (1998), Blinov (2000), Onipko (2002), Nikolaychuk and Zubitskaya (2003), Dremova et al. (2003), Nazina (2006), Bogdanova and Bashkirova (2010), Maznev (2014)
4.44	*Cichorium intybus* L*.* leaves; *Fragaria vesca* L*.* leaves; *Polygonum aviculare* L*.* aerial part; *Taraxacum campylodes* G.E.Haglund. leaves; (4:3:2:3)	Infusion; 1 table spoon in 200 ml of boiling water	66 ml a day before eating	Diabetes	Sinyakov (1992), Sinyakov (1999), Efimov and Shcherbak (1993), Nikultseva (1994), Nikolaychuk (1997), Tarasenko et al. (1998), Blinov (2000); Podduev (2001), Dremova et al. (2003), Nazina (2006), Korodetsky (2006), Ryzhenko (2007), Bogdanova and Bashkirova (2010)
4.45	*Mentha × piperita* L*.* leaves; *Ribes nigrum* L. leaves; *Rubus caesius* L. leaves; *Taraxacum campylodes* G.E.Haglund. leaves; (1:3:2:4)	Infusion; 1 table spoon in 200 ml of boiling water	2–3 table spoons 3 times a day before eating	Diabetes	Nikultseva (1994), Nikolaychuk (1997), Sinyakov (1999), Blinov (2000), Nazina (2006), Ryzhenko (2007), Davydovich et al. (2008), Maznev (2014)
4.46	*Helichrysum arenarium* (L.) Moench flowers; *Rosa majalis* Herrm. fruits; *Vaccinium myrtillus* L. leaves; *Zea mays* L. corn silk; (1:2:5:2)	Infusion; 2 table spoons in 300 ml of boiling water, maceration 12 h in thermos	66 ml 3–4 times a day 30 min before eating	Diabetes	Sinyakov (1992), Sinyakov (1999), Efimov and Shcherbak (1993), Tarasenko et al. (1998), Fedyukovich (1998), Blinov (2000)
4.47	*Alchemilla xanthochlora* Rothm. aerial part; *Juniperus communis* L. fruits; *Linum usitatissimum* L. seeds; *Vaccinium myrtillus* L. leaves; (1:2:2:4)	Decoction; 1table spoon in 200 ml of water	200 ml 2–3 times a day	Diabetes	Yordanov et al. (1972), Matkovskaya et al. (1988); Chirkov and Seryi (1993); Efimov and Shcherbak (1993); Dremova et al. (2003); Davydovich et al. (2008); Bogdanova and Bashkirova (2010); Volynchenko(2003); Maznev (2014); Pigulevskaya (2018)
4.48	*Galega officinalis* L. aerial part; *Mentha × piperita* L. leaves; *Phaseolus vulgaris* L. pericarp; *Zea mays* L. corn silk; (1:1:7:2)	Infusion; 3 table spoons in 400 ml of boiling water	100 ml 3 times a day	Diabetes	Chirkov and Seryi (1993); Efimov and Shcherbak (1993); Bogdanova and Bashkirova (2010)
4.49	*Betula* spp.^c^leaves; *Frangula alnus* Mill. bark; *Vaccinium myrtillus* L. leaves; *Vaccinium vitis-idaea* L*.* leaves; (1:1:2:2)	Decoction; *Frangula* bark cut, boil 20 min in boiling water + Infusion; in 300 ml boiling water and boil 3 min	100 ml 2–3 times a day before eating	Diabetes	Ryzhenko (2007)
4.50	*Taraxacum campylodes* G.E.Haglund. leaves; *Urtica dioica* L*.* leaves; *Vaccinium myrtillus* L. leaves; *Vaccinium vitis-idaea* L. leaves; (1:1:1:1)	Infusion; 1 table spoon in 300 ml of boiling water	100 ml 2–3 times a day 20 min before eating	Diabetes	Ryzhenko, (2007)
4.51	*Avena sativa* L. aerial part & straw; *Betula pendula* Roth*.* leaves; *Linum usitatissimum* L. seeds; *Vaccinium myrtillus* L. leaves; (1:1:1:1)	Infusion; 3 table spoons in 600 ml of boiling water, boil 10 min, maceration 30–40 min	50 ml 6–8 times a day 20–30 min before eating	Diabetes	Podduev (2001)
4.52	*Avena sativa* L. aerial part & straw; *Equisetum arvense* L. aerial part; *Linum usitatissimum* L. seeds; *Oplopanax elatus* (Nakai) Nakai roots and rhizomes; (2:2:2:1)	Infusion; 1 table spoon in 200 ml of boiling water, boil 15 min, maceration 45 min	100 ml a day	Diabetes	Podduev (2001)
4.53	*Arctostaphylos uva-ursi* (L.) Spreng. leaves; *Avena sativa* L. aerial part & straw; *Linum usitatissimum* L. seeds; *Phaseolus vulgaris* L*.* pericarp; (1:1:1:1)	Infusion; 1 table spoon in 200 ml of boiling water, boil 10 min, maceration 2 h	200 ml a day	Diabetes	Podduev (2001)
4.54	*Juglans regia* L. leaves; *Mentha × piperita* L*.* leaves; *Polygonatum odoratum* (Mill.) Druce leaves; *Polygonum aviculare* L. aerial part; (3:2:2:3)	Infusion; 2 table spoons in 500 ml of boiling water, boil 2–3 min, maceration 30–40 min	100 ml 3–4 times a day 30 min before eating	Diabetes	Podduev (2001)
4.55	*Aralia elata* (Miq.) Seem roots; *Galega officinalis* L. aerial part; *Rosa majalis* Herrm. fruits; *Vaccinium myrtillus* L. fruits; (2:3:2:3)	Infusion or decoction; 10 g in 400 ml of water	66–100 ml 3 times a day	Lowering of blood glucose level	Sokolov (2000)
4.56	*Centaurium erythraea* Rafn aerial part; *Solanum tuberosum* L. juice; *Vaccinium myrtillus* L. leaves; *Viburnum opulus* L. berries juice; (1:3:4:2)	Infusion; 50 g in 1,000 ml of boiling water, maceration 10–12 h at room temp.	50–66 ml (warm) 3–4 times a day before eating	Diabetes accompanied by gastritis	Brusenskaya and Kaz’min, (2005)
4.57	*Cichorium intybus* L. roots; *Rosa majalis* Herrm*.* fruits; *Schisandra chinensis* (Turcz.) Baill. leaves; *Taraxacum campylodes* G.E.Haglund. roots; (3:4:1:3)	Infusion; 1 tea spoon in boiling water	2 table spoons 4 times a day before eating and 30 min before sleeping for 30 days	Diabetes accompanied by impotence in men	Brusenskaya and Kaz’min, (2005)
4.58	*Betula pendula* Roth*.* leaves; *Ribes nigrum* L. leaves; *Rubus caesius* L. leaves; *Trifolium pratense* L. leaves; (1:1:1:1)	Infusion; 1 table spoon in 300 ml of boiling water	100 ml with 1/2 tea spoon of honey 3 times a day before eating	Diabetes accompanied by impotence in men	Brusenskaya and Kaz’min, (2005)
4.59	*Alchemilla xanthochlora* Rothm. aerial part; *Phaseolus vulgaris* L. pericarp; *Taraxacum campylodes* G.E.Haglund. roots; *Vaccinium myrtillus* L. leaves; (1:1:1:1)	Infusion; 1 table spoon in 200 ml of water	1 table spoon 3 times a day	Diabetes accompanied by impotence in men	Brusenskaya and Kaz’min, (2005)
4.60	*Achillea millefolium* L. aerial part; *Arctium* spp.^b^ roots; *Helichrysum arenarium* (L.) Moench flowers; *Hypericum* spp*.* ^e^ aerial part; (1:35:1:8)	Infusion; 2 table spoons in 300–400 ml of water	100 ml morning and evening	Diabetes accompanied by liver and gallbladder diseases	Brusenskaya and Kaz’min, (2005)
4.61	*Crataegus* spp.^f^ lowers; *Crataegus* spp.^f^fruits; *Leonurus* spp. ^d^leaves; *Vaccinium myrtillus* L. leaves; (1:1:1:4)	Infusion; boiling of *Crataegus* fruits 20 min, than maceration of 30 g of mixture in boiling water 8–12 h at room tempature	100 ml 3 times a day before eating	Diabetes accompanied by angina and shortness of breath	Brusenskaya and Kaz’min, (2005)
4.62	*Asperula graveolen*s M.Bieb. ex Schult. & Schult.f. aerial part; *Fragaria vesca* L*.* leaves; *Melissa officinalis* L. leaves; *Thymus serpyllum* L. aerial part; (1:2:1:1)	Infusion (herbal tea); 1 table spoon in 200 ml of boiling water	100 ml 4–5 times a day	Metabolism improving	Chirkov and Seryi (1993)
4.63	*Betula pendula* Roth. leaves; *Prunus spinosa* L. flowers; *Sambucus nigra* L. flowers; *Urtica dioica* L. leaves; (1:1:1:1)	Infusion (herbal tea); 1 table spoon in 200 ml of boiling water	200–400 ml a day, before first eating	Metabolism improving and diuretics	Chirkov and Seryi (1993)
4.64	*Frangula alnus* Mill. bark; *Glycyrrhiza glabra* L*.* roots; *Viola tricolor* L. aerial part; *Juglans regia* L. leaves; (1:1:4:4)	Decoction; 1 table spoon in 600 ml of water	400 ml a day	Exudative diathesis caused by metabolic disorder	Chirkov and Seryi (1993)
4.65	*Betula pendula* Roth. leaves; *Melissa officinalis* L. leaves; *Salvia officinalis* L. leaves; *Urtica dioica* L. leaves; (1:1:1:1)	Infusion (herbal tea); 1 table spoon in 200 ml of boiling water	200 ml in the morning and 200 ml in the evening	Acne, in case of metabolic disorder	Chirkov and Seryi (1993)
4.66	*Betula pendula* Roth*.* leaves; *Frangula alnus* Mill. bark; *Linum usitatissimum* L. seeds; *Urtica dioica* L. leaves; (1:1:1:1)	Decoction; 1 table spoon in 200 ml of water	66 ml 3 times a day	Skin rash, metabolic disorder	Chirkov and Seryi (1993)
**5 plants**					
5.1	*Foeniculum vulgare* Mill. fruits; *Frangula alnus* Mill. bark; *Mentha × piperita* L*.* leaves; *Petroselinum crispum* (Mill.) Fuss fruits; *Taraxacum campylodes* G.E.Haglund. roots; (1:3:1:1:1)	Infusion; 2 table spoons in 500 ml of boiling water	500 ml in the morning before eating	Obesity	Yordanov et al. (1972); Lager, (1991); Sinyakov (1992); Chirkov and Seryi (1993); Efimov and Shcherbak (1993); Kukes (1999); Dontsov and Dontsov (2000); Sokolov (2000); Bubenchikova et al.(2003); Podduev (2001); Maznev (2005); Safonov (2016)
5.2	*Achillea millefolium* L. aerial part; *Frangula alnus* Mill. bark; *Juniperus communis* L. fruits; *Levisticum officinale* W.D.J.Koch roots; *Phaeophyceae* (*Cystoseira barbata* (Stackh.) C.Agardh) thallus; (10:3:1:1:3)	Decoction; 2 table spoons in 500 ml of water	100 ml 3–4 times a day	Obesity	Yordanov et al. (1972); Efimov and Shcherbak (1993); Chirkov and Seryi (1993)
5.3	*Frangula alnus* Mill. bark; *Levisticum officinale* W.D.J.Koch roots; *Ononis spinosa* L. roots; *Phaeophyceae* (*Cystoseira barbata* (Stackh.) C.Agardh) thallus; *Taraxacum campylodes* G.E.Haglund. roots; (5:1:1:2:1)	Decoction; 2 table spoons in 500 ml of water	132–200 ml 2–3 times a day	Obesity	Chirkov and Seryi (1993)
5.4	*Matricaria chamomilla* L. flowers; *Foeniculum vulgare* Mill. fruits; *Mentha × piperita* L. leaves; *Sambucus nigra* L. flowers; *Tilia cordata* Mill. flowers; (1:1:1:1:1)	Infusion; 10 g in 200 ml of boiling water	200 ml 2–3 times a day	Obesity	Sokolov and Zamotaiev (1984); Lager (1991); Sinyakov (1992); Efimov and Shcherbak (1993); Lager (2002); Tarasenko et al. (1998); Kukes (1999); Sokolov (2000); Dontsov and Dontsov (2000); Said-Shah (2001); Podduev (2001); Maznev (2005); Kiyanova (2005); Rendiuk (2006)
5.5	*Arctostaphylos uva-ursi* (L.) Spreng. leaves; *Frangula alnus* Mill. bark; *Linum usitatissimum* L. seeds; *Panax ginseng* C.A.Mey roots; *Taraxacum campylodes* G.E.Haglund. roots; (3:4:5:4:4)	Infusion; 10 g in 400 ml of water.	66–100 ml 3 times a day	Obesity	Sokolov (2000)
5.6	*Foeniculum vulgare* Mill. fruits; *Hypericum perforatum* L. aerial part; *Mentha × piperita* L. leaves; *Sambucus nigra* L. flowers *Tilia cordata* Mill. flowers; (4:4:3:3:4)	Infusion	2 table spoons 3 times a day 30 min before eating for 1–1.5 months	Obesity	Kukes (1999)
5.7	*Betula* spp*.* ^c^ leaves; *Elymus repens*(L.) Couldrhizomes; *Frangula alnus* Mill. bark; *Melissa officinalis* L*.* aerial part; *Taraxacum campylodes* G.E.Haglund. roots; (2:2:1:1:1)	Infusion; 2 table spoons in 500 ml of boiling water, maceration 12 h in thermos	100 ml 3 times a day	Obesity	Osetrov (1993); Osetrov and Shreter (2001)
5.8	*Achillea millefolium* L. aerial part; *Carum carvi* L. fruits; *Frangula alnus* Mill. bark; *Viola tricolor* L. aerial part; *Zea mays* L. corn silk; (1:1:6:1:1)	Infusion; 4 table spoons in 800 ml of boiling water	400 ml 2 times a day	Obesity	Lager (1991), Lager (2002); Efimov and Shcherbak (1993); Kukes (1999); Dontsov and Dontsov (2000); Bubenchikova et al. (2003)
5.9	*Humulus lupulus* L. fruits; *Panax ginseng* C.A.Mey roots; *Phaseolus vulgaris* L. pericarp; *Rosa majalis* Herrm. fruits; *Sorbus aucuparia* L*.* fruits; (3:3:5:4:5)	Infusion; 10 g in 400 ml of water	66–100 ml 3 times a day	Lowering of blood glucose level	Sokolov (2000)
5.10	*Galega officinalis* L. aerial part; *Vaccinium myrtillus* L. leaves; *Phaseolus vulgaris* L. pericarp; *Taraxacum campylodes* G.E.Haglund. roots; *Urtica dioica* L. leaves; (1:1:1:1:1)	Infusion; 1 table spoon in 200 ml of boiling water	200 ml 3–4 times a day before eating	Diabetes (early stages)	Yordanov et al. (1972); Sinyakov (1992); Chirkov and Seryi (1993); Efimov and Shcherbak (1993); Nikultseva (1994); Dmitriev et al. (1994); Nikolaychuk (1997); Fedyukovich (1998); Nikolaychuk and Zubitskaya (2003); Ryzhenko (2007); Lavrenova and Lavrenov (2007); Davydovich et al. (2008); Bogdanova and Bashkirova (2010); Ruzhenkova (2014); Maznev (2014)
5.11	*Juglans regia* L. leaves; *Phaseolus vulgaris* L. pericarp; *Portulaca oleracea* L. leaves; *Ribes nigrum* L. leaves; *Vaccinium myrtillus* L. leaves; (2:3:2:3:2)	Herbal tea; 3 table spoons in 1,000 ml of boiling water	100 ml 7 times a day	Diabetes	Protasenya and Vasilenko (1992)
5.12	*Equisetum arvense* L. aerial part; *Hypericum* spp*.* ^e^flowers; *Phaseolus vulgaris* L. pericarp; *Polygonum aviculare* L. aerial part; *Vaccinium myrtillus* L. leaves; (1:4:4:1:4)	Infusion; 70 g in 1,000 ml of water, boil 2 min, maceration 8–12 h in a dark place	66 ml 3–4 times a day before eating	Diabetes	Brusenskaya and Kaz’min (2005)
5.13	*Galega officinalis* L. aerial part; *Phaseolus vulgaris* L. pericarp; *Taraxacum campylodes* G.E.Haglund. roots; *Taraxacum campylodes* G.E.Haglund. leaves; *Vaccinium myrtillus* L. leaves; (4:4:3:2:5)	Infusion; 1 table spoon in 200 ml of boiling water	66–100 ml 3–4 times a day before eating	Diabetes	Brusenskaya and Kaz’min (2005)
5.14	*Hypericum* spp. ^e^flowers; *Inula helenium* L. roots; *Sambucus nigra* L. leaves; *Taraxacum campylodes* G.E.Haglund. leaves; *Urtica dioica* L. leaves; (2:1:2:2:1)	Infusion (herbal tea); 1 table spoon in 200 ml of boiling water	100 ml 2 times a day before eating	Diabetes	Brusenskaya and Kaz’min (2005)
5.15	*Arctium lappa* L. roots; *Galega officinalis* L. aerial part; *Oenanthe aquatica* (L.) Poir. fruits; *Polygonum aviculare* L*.* aerial part; *Symphytum officinale* L. root; (4:7:3:3:3)	Infusion; 2 table spoons in 500 ml of boil water	200 ml 2–3 times a day before eating	Diabetes	Yordanov et al. (1972); Efimov and Shcherbak (1993); Sinyakov (1999); Davydovich et al. (2008); Bogdanova and Bashkirova (2010)
5.16	*Galega officinalis* L. aerial part; *Taraxacum campylodes* G.E.Haglund. leaves; *Urtica dioica* L. leaves; *Vaccinium myrtillus* L. leaves; *Vaccinium vitis-idaea* L. leaves; (1:1:1:1:1)	Infusion; 2 table spoons in 500 ml of boiling water, boil 5–7 min, maceration 1–2 h at room tempature	100 ml 2–3 times a day 20 min before eating	Diabetes	Sinyakov (1992); Efimov and Shcherbak (1993); Nikultseva (1994); Nikolaychuk (1997); Tarasenko et al. (1998); Fedyukovich (1998); Blinov (2000); Podduev (2001); Nikolaychuk and Zubitskaya (2003); Nazina (2006); Korodetsky (2006); Davydovich et al. (2008); Bogdanova and Bashkirova (2010); Maznev (2014)
5.17	*Galega officinalis* L. aerial part; *Phaseolus vulgaris* L. pericarp; *Taraxacum campylodes* G.E.Haglund. roots; *Urtica dioica* L. leaves; *Vaccinium myrtillus* L. leaves; (1:1:1:1:1)	Infusion; 1 table spoon in 200 ml of boiling water	66 ml 3–4 times a day before eating	Diabetes	Matkovskaya et al. (1988); Blinov (2000); Nazina (2006); Pigulevskaya (2018)
5.18	*Betula* spp. ^c^leaves; *Frangula alnus* Mill. bark; *Galega officinalis* L. aerial part; *Vaccinium myrtillus* L. leaves; *Vaccinium vitis-idaea* L*.* leaves; (1:1:2:2:2)	Infusion; 7 g in 300 ml of boiling water + Decoction; *Frangula* bark cut in 300 ml of boil warter, boil 20 min	66 ml before each eating	Diabetes	Sinyakov (1992); Efimov and Shcherbak (1993); Nikultseva (1994); Nikolaychuk (1997); Tarasenko et al. (1998); Fedyukovich (1998); Blinov (2000); Podduev (2001); Nikolaychuk and Zubitskaya (2003); Nazina (2006); Davydovich et al. (2008); Bogdanova and Bashkirova (2010); Maznev (2014)
5.19	*Cichorium intybus* L*.* leaves; *Galega officinalis* L. aerial part; *Juglans regia* L*.* leaves; *Taraxacum campylodes* G.E.Haglund. leaves; *Urtica dioica* L*.* leaves; (1:1:1:1:1)	Infusion; 1 table spoon in 400 ml of boiling water	2–3 table spoons 3 times a day 15–20 min before eating	Diabetes	Sinyakov (1992); Efimov and Shcherbak (1993); Nikultseva (1994); Nikolaychuk (1997); Fedyukovich (1998); Blinov (2000); Nikolaychuk and Zubitskaya (2003); Dremova et al. (2003); Nazina (2006); Ryzhenko (2007); Bogdanova and Bashkirova (2010)
5.20	*Elymus repens* (L.) Gould rhizomes; *Sambucus nigra* L. flowers; *Tilia cordata* Mill. flowers; *Tussilago farfara* L. leaves; *Verbascum densiflorum* Bertol. flowers; (1:1:1:1:1)	Decoction; 5 table spoons in 600 ml of water	66 ml 5–6 times a day	Diabetes	Chirkov and Seryi (1993)
5.21	*Althaea officinalis* L. roots; Centaurium erythraea Rafn aerial part;Mentha × piperita L. aerial part; *Prunus* *avium*(L.) L. shoots; *Zea mays* L. corn silk; (1:1:5:1:1)	90 g in 2000 ml of boiling water; evaporate to residue of 1,000 ml	150 ml in the morning before eating, than 1 table spoon every 2 h during the day	Diabetes	Osetrov (1993); Osetrov and Shreter (2001)
5.22	*Arctostaphylos uva-ursi* (L.) Spreng. leaves; *Mentha × piperita* L*.* leaves; *Ribes nigrum* L. leaves; *Rubus caesius* L. leaves; *Vaccinium myrtillus* L. leaves; (1:1:1:1:1)	Infusion; 1 table spoon in 200 ml of boiling water, maceration 30 min	100 ml 3 times a day	Diabetes	Efimov and Shcherbak (1993); Nikolaychuk (1997); Fedyukovich (1998); Smolianskii and Lifliandskii (2004); Bogdanova and Bashkirova (2010); Melik-Gusseinov and Rekkandt (2014)
5.23	*Arctium* spp*.* ^b^roots; *Juglans regia* L. leaves; *Phaseolus vulgaris* L. pericarp; *Sambucus nigra* L. flowers or roots; *Vaccinium myrtillus* L. leaves; (1:1:1:1:1)	Infusion; 100 g in 400 ml of boiling water, maceration 5 h	100 ml 3 times a day after eating	Diabetes	Smolianskii and Lifliandskii (2004); Pigulevskaya (2018)
5.24	*Equisetum arvense* L. aerial part; *Hypericum perforatum* L. aerial part; *Taraxacum campylodes* G.E.Haglund. roots; *Urtica dioica* L. leaves; *Vaccinium myrtillus* L. leaves; (1:1:1:1:1)	Infusion; 1 table spoon in 200 ml of boiling water, maceration 30 min	66 ml 3 times a day before eating	Diabetes	Efimov and Shcherbak (1993); Nikolaychuk (1997); Fedyukovich (1998); Nikolaychuk and Zubitskaya (2003); Smolianskii and Lifliandskii (2004); Bogdanova and Bashkirova (2010)
5.25	*Cichorium intybus* L. roots; *Crataegus* spp.^f^fruits; *Elymus* *repens* *(*L.*)* Gould rhizomes; *Rosa* spp.^g^fruits; *Vaccinium myrtillus* L. fruits; (3:2:3:2:2)	Infusion; 1 table spoon in 200 ml of boiling water, boil 10 min, maceration at room tempature	100 ml 4 times a day 30 min before eating.	Diabetes	Efimov and Shcherbak (1993); Rendiuk (2006); Bogdanova and Bashkirova (2010)
5.26	*Sambucus nigra* L. flowers; *Taraxacum campylodes* G.E.Haglund. roots *Urtica dioica* L. leaves; *Vaccinium myrtillus* L. fruits; *Vaccinium myrtillus* L. leaves; (3:3:4:4:4)	Infusion	1–2 table spoon 3 times a day 30 min before eating for 1–1.5 months.	Diabetes	Kukes (1999)
5.27	*Arctium lappa* L. roots; *Phaseolus vulgaris* L. pericarp; *Urtica dioica* L. leaves; *Vaccinium myrtillus* L. leaves; *Vaccinium vitis-idaea* L. leaves; (1:1:1:1:1)	Infusion; 20 g in 200 ml of boiling water	66 ml 3 times a day	Diabetes	Lager (1991), Lager (2002)
5.28	*Cichorium intybus* L. roots; *Hypericum perforatum* L. aerial part; *Mentha × piperita* L*.* leaves; *Taraxacum campylodes* G.E.Haglund. roots; *Vaccinium myrtillus* L. leaves; (1:1:1:1:1)	Infusion; 1 table spoon in 300 ml of boiling water	66 ml 3 times a day	Diabetes	Lager (1991), Lager (2002); Efimov and Shcherbak (1993); Nikolaychuk (1997); Fedyukovich (1998); Nikolaychuk and Zubitskaya (2003); Bogdanova and Bashkirova (2010); Maznev (2014); Pigulevskaya (2018)
5.29	*Cichorium intybus* L*.* leaves; *Galega officinalis* L. aerial part; *Juglans regia* L. leaves; *Taraxacum campylodes* G.E.Haglund. leaves; *Urtica dioica* L*.* leaves; (1:1:1:1:1)	Infusion; 2 table spoons in 500 ml of boiling water, boil 2–3 min, maceration 30–40 min	50 ml 3–4 times a day 15–20 min before eating	Diabetes	Sinyakov (1999)
5.30	*Galega officinalis* L. aerial part; *Phaseolus vulgaris* L. pericarp; *Taraxacum campylodes* G.E.Haglund. roots; *Urtica dioica* L. leaves; *Vaccinium myrtillus* L. leaves; (1:1:1:1:1)	Infusion; 2 table spoons in 500 ml of boiling water, maceration 12 h in thermos	100 ml (warm) 3 times a day 30 min before eating	Diabetes	Sinyakov (1999)
5.31	*Betula pendula* Roth*.* leaves; *Frangula alnus* Mill. bark; *Galega officinalis* L. aerial part; *Vaccinium myrtillus* L. leaves; *Vaccinium vitis-idaea* L*.* leaves; (3:2:5:5:5)	Decoction; *Frangula* bark boil for 20 min. Infusion; other part in 500 ml of boiling water, boil for 3–4 min, maceration 30 min. Mix with frangula decoction.	66–100 ml 2–3 times a day 20–30 min before eating	Diabetes	Sinyakov (1999)
5.32	*Arctium lappa* L. roots; *Cichorium intybus* L. roots; *Linum usitatissimum* L. seeds; *Phaseolus vulgaris* L. pericarp; *Vaccinium myrtillus* L. leaves; (2:2:2:7:7)	Infusion; 2–3 table spoons in 500 ml water, maceration 12 h, 15 min in boil water bath, maceration 1 h	200 ml 3–4 times a day 30 min before eating	Diabetes	Sinyakov (1999)
5.33	*Alchemilla xanthochlora* Rothm. roots and aerial part*; Phaseolus vulgaris* L. pericarp; *Taraxacum campylodes* G.E.Haglund. roots; *Urtica dioica* L. leaves; *Vaccinium myrtillus* L. leaves (1:1:1:1:1)	Infusion; 1 table spoon in 200 ml of water	1 table spoon 3 times a day	Diabetes	Podduev (2001)
5.34	*Angelica archangelica* L. aerial part; *Betula spр.* Leaves; *Frangula alnus* Mill. bark; *Vaccinium myrtillus* L. leaves; *Vaccinium vitis-idaea* L*.* leaves; (2:1:1:2:2)	Decoction; *Frangula* bark cut boil 20 min + Infusion; other part in 300 ml of boiling water, 3 min boil. Mix with frangula decoction.	70 ml 2–3 times a day before eating	Diabetes	Podduev (2001)
5.35	*Avena sativa* L. aerial part; *Fagopyrum esculentum* Moench flowers; *Linum usitatissimum* L. seeds; *Prunus laurocerasus* L. leaves; *Sambucus ebulus* L. flowers; (3:2:2:3:2)	Herbal tea; 3 table spoons in 800 ml of boiling water	50 ml 6 times a day	Diabetes. In case of severe condition of the disease course	Protasenya and Vasilenko (1992)
5.36	*Acorus calamus* L. root; *Arctium* spp*.* ^b^leaves; *Matricaria chamomilla* L. flowers; *Frangula alnus* Mill. bark; *Vaccinium myrtillus* L. leaves; (1:3:2:1:4)	Infusion; 55 g in 1,000 ml of boiling water, maceration 10–12 h at room tempature	3 table spoons 20–30 min before eating	Diabetes accompanied by colitis and constipation	Brusenskaya and Kaz’min (2005)
5.37	*Arctium lappa* L. roots; *Cichorium intybus* L. roots; *Rubus caesius* L. root; *Valeriana officinalis* L. roots and rhizomes; *Vincetoxicum hirundinaria* Medik. roots, rhizomes, leaves and seeds; (2:3:1:3:3)	Herbal tea; 3 table spoons in 1,000 ml of boiling water	100 ml 7 times a day	Diabetes accompanied by metabolic polyarthritis, rheumatoid arthritis	Protasenya and Vasilenko (1992)
5.38	*Arctium lappa* L*.* roots; *Equisetum arvense* L. aerial part; *Gratiola officinalis* L. aerial part; *Orthosiphon aristatus* (Blume) Miq. leaves; *Phaseolus vulgaris* L. pericarp; (2:3:2:1:4)	Herbal tea; 3 table spoons in 1,000 ml of boiling water	70 ml 6 times a day	Diabetes accompanied by edema related to renal failure	Protasenya and Vasilenko (1992)
5.39	*Asparagus officinalis* L. rhizomes and aerial part; *Centaurium erythraea* Rafn aerial part; *Fraxinus excelsior* L. leaves *Oplopanax elatus* (Nakai) Nakai roots and rhizomes; *Plantago major* L. leaves; (2:2:3:2:3)	Herbal tea; 3 table spoons in 800 ml of boiling water	50 ml 6 times a day	Diabetes accompanied by chronic gastritis with reduced secretory function	Protasenya and Vasilenko (1992)
5.40	*Equisetum arvense* L. aerial part; *Gnaphalium uliginosum* L. aerial part; *Rosa majalis* Herrm. fruits; *Sambucus nigra* L. flowers; *Syringa vulgaris* L. buds; (3:3:2:2:2)	Herbal tea; 3 table spoons in 1,200 ml of boiling water	100 ml 6 times a day	Diabetes accompanied by hypertension and slight edema of the lower extremities	Protasenya and Vasilenko (1992)
5.41	*Centaurium erythraea* Rafn aerial part; *Cichorium intybus* L. roots; *Hypericum* spp*.* ^e^flowers; *Juglans regia* L. leaves; *Plantago major* L. leaves; (1:2:4:1:3)	Decoction; *Cichorium* roots in 100 ml of water + Infusion; 45 g other part in 1,000 ml of boil water, maceration 3–5 h. Mix with cichorium decoction	50 ml 3 times a day before eating	Diabetes accompanied by colitis and constipation	Efimov and Shcherbak (1993); Brusenskaya and Kaz’min (2005); Davydovich et al. (2008); Bogdanova and Bashkirova (2010)
5.42	*Alnus spp.* (*A. incana* (L.) Moench and *A. glutinosa* (L.) Gaertn*.)* fruits; *Centaurium erythraea* Rafn aerial part; *Mentha × piperita* L. leaves; *Quercus* spp.^h^bark; *Vaccinium myrtillus* L. leaves; (2:1:1:4:4)	Infusion; 60 g in 1,000 ml of water, maceration 3–4 h at room tempature	50 ml 3–4 times a day before eating, for 7–10 days	Diabetes with frequent diarrhea	Brusenskaya and Kaz’min (2005)
5.43	*Betula* spp*.* ^c^sap; *Daucus* *sativus*Roehl. juice; *Leonurus* spp ^d^leaves; *Phaseolus vulgaris* L. pericarp; *Viburnum opulus* L. berries juice; (20:2:1:4:2)	Infusion; 40 g in 1,000 ml of boil water, maceration 3–5 h in a dark place	100 ml 4–6 times a day before eating	Diabetes accompanied by angina and shortness of breath	Brusenskaya and Kaz’min (2005)
5.44	*Polygonum aviculare* L*.* aerial part *Zea mays* L. corn silk; *Hypericum* spp*.* ^e^ flowers; *Viburnum opulus* L. berries; *Arctostaphylos uva-ursi* (L.) Spreng*.* leaves; (1:2:2:1:2)	Infusion; 40 g in 1,000 ml of boil water, maceration 3–5 h in a dark place	100 ml 3–4 times a day after eating	Diabetes accompanied by kidney and bladder disease	Brusenskaya and Kaz’min (2005)
5.45	*Anethum graveolens* L. fruits; *Mentha × piperita* L. leaves; *Crataegus sanguinea* Pall. flowers; *Helichrysum arenarium* (L.) Moench*.* flowers; *Matricaria chamomilla L.* flowers; (3:3:2:2:2)	Infusion (herbal tea); 1 table spoon in 200 ml of boiling water	100 ml 3 times a day 1 h after eating	Diabetes accompanied by chronic pancreatitis	Chirkov and Seryi (1993); Kukes (1999); Bubenchikova et al. (2003)
5.46	*Taraxacum campylodes G.E.Haglund.* roots; *Arctium lappa* L*.* roots; *Rubia tinctorum* L. roots; *Saponaria officinalis* L. roots or *Bidens tripartita* L. aerial part; *Glycyrrhiza glabra* L. roots; (3:3:6:6:2)	Decoction; 1 table spoon in 200 ml of water	200–400 ml a day before first eating	Exudative diathesis caused by metabolic disorder	Chirkov and Seryi, (1993)
5.47	*Humulus lupulus* L. fruits; *Menyanthes trifoliata* L. leaves*; Gentiana lutea* L. root*; Melissa officinalis* L. aerial part*; Achillea millefolium* L. aerial part; (2:3:2:4:1)	Herbal tea; 3 table spoons in 1,000 ml of boiling water	100 ml 6 times a day	Metabolic disorder with multiple skin furuncles	Protasenya and Vasilenko (1992)
**6 plants**					
6.1	*Achillea millefolium* L. aerial part; *Carum carvi* L. fruits; *Frangula alnus* Mill. bark; *Prunus spinosa* L. flowers; *Viola tricolor* L. aerial part; *Zea mays* L. corn silk; (1:1:6:1:1:1)	Decoction; 2 table spoons in 400 ml of water	400 ml 2 times a day	Obesity	Chirkov and Seryi (1993)
6.2	*Arctium* spp*.* ^b^roots; *Urtica dioica* L. leaves; *Avena sativa* L. aerial part; *Vaccinium myrtillus* L. leaves; *Fragaria vesca* L. leaves; *Rosa spp.* ^g^fruits (3:4:4:4:3:4)	Herbal tea; 1 table spoon in 200 ml of boiling water	100 ml 2 times a day before eating	Diabetes prevention	Brusenskaya and Kaz’min (2005)
6.3	*Vaccinium myrtillus* L. leaves; *Rosa* spp*.* ^g^fruits; *Phaseolus vulgaris* L. pericarp; *Fragaria vesca* L. leaves; *Taraxacum campylodes* G.E.Haglund. roots; *Achillea millefolium* L. aerial part; (4:5:4:3:2:1)	Herbal tea; 1 table spoon in 200 ml of boiling water	100 ml 2 times a day before eating	Diabetes prevention	Brusenskaya and Kaz’min (2005); Maznev (2014)
6.4	*Avena sativa* L. aerial part; *Cichorium intybus* L. roots; *Galega officinalis* L. aerial part; *Linum (usitatissimum* L.*)* seeds; *Phaseolus vulgaris* L. pericarp; *Vaccinium myrtillus* L. leaves; (1:1:1:1:1:1)	Infusion; 10 g in 300 ml of boiling water, maceration 2 h	100 ml 3 times a day before eating	Insulin-dependent diabetes	Kolesova et al. (1998)
6.5	*Urtica dioica* L. leaves; *Arctium lappa* L*.* roots; *Linum usitatissimum* L. seeds; *Juniperus communis* L. fruits; *Taraxacum campylodes* G.E.Haglund. roots; *Vaccinium myrtillus* L. leaves; (2:2:1:1:1:3)	Infusion; 3 table spoon in 600 ml of boiling water	200 ml 2–3 times a day before eating	Diabetes	Efimov and Shcherbak (1993); Fedyukovich (1998); Dontsov and Dontsov (2000); Bogdanova and Bashkirova (2010)
6.6	*Linum usitatissimum* L. seeds; *Vaccinium vitis-idaea* L. leaves; *Inula helenium* L. roots; *Gnaphalium uliginosum* L*.* aerial part; *Zea mays* L. corn silk; *Matricaria chamomilla L.* flowers; (4:4:3:3:3:3)	Infusion; 3 table spoons in 500 ml of boiling water, maceration 12 h in thermos	130–140 ml (warm) 3 times a day 20–30 min before eating	Diabetes	Sinyakov (1992), Sinyakov (1999); Tarasenko et al. (1998); Podduev (2001)
6.7	*Mentha × piperita* L*.* aerial part; *Rosa* spp. ^g^fruits; *Sorbus aucuparia* L. fruits; *Taraxacum campylodes* G.E.Haglund. roots; *Urtica dioica* L. leaves*; Vaccinium myrtillus* L. shoots; (1:1:1:1:1:1)	Infusion; 6 g in 350 ml of boiling water, 10 min in boil water bath, maceration 3 h in thermos	100 ml 3 times a day before eating.	Diabetes	Vinogradova et al. (2001)
6.8	*Arctium* spp*.* ^b^roots; *Equisetum arvense* L. aerial part; *Fragaria vesca* L. leaves; *Mentha × piperita* L*.* aerial part; *Vaccinium myrtillus* L. shoots; *Vaccinium vitis-idaea* L. leaves; (1:1:1:1:1:1)	Infusion; 8 g in 300 ml of boiling water, boil 2 min, maceration 2 h in thermos	50–70 ml (warm) 10 min before eating	Diabetes	Vinogradova et al. (2001)
6.9	*Betula* spp*.* ^c^leaves; *Foeniculum vulgare* Mill. fruits; *Mentha × piperita* L*.* aerial part; *Petroselinum crispum* (Mill.) Fuss aerial part; *Ribes nigrum* L. leaves; *Rosa* spp. ^g^fruits; (1:1:1:1:1:1)	Infusion; 8 g in 300 ml of boiling water, 15 min in boil water bath, maceration 1 h in thermos	66–100 ml 3–4 times a day before eating	Diabetes	Vinogradova et al. (2001)
6.10	*Galega officinalis* L. aerial part; *Laurus nobilis* L. leaves; *Mentha × piperita* L*.* aerial part; *Phaseolus vulgaris* L. pericarp; *Sorbus aucuparia* L. fruits; *Vaccinium myrtillus* L. leaves; (1:1:1:1:1:1)	Infusion; 8 g in 300 ml of boiling water, 15 min in boil water bath, maceration 2 h in thermos	66–100 ml 3–4 times a day before eating	Diabetes	Vinogradova et al. (2001)
6.11	*Glycyrrhiza glabra* L. roots; *Hypericum* spp*.* ^e^aerial part; *Juglans regia* L. leaves; *Phaseolus vulgaris* L. pericarp; *Syringa vulgaris* L. buds; *Vaccinium vitis-idaea* L*.* leaves; (1:1:1:1:1:1)	Infusion; 8 g in 400 ml of boiling water, 15 min in boil water bath, maceration 2 h in thermos	100 ml (warm) 3 times a day10 min before eating	Diabetes	Vinogradova et al. (2001)
6.12	*Cichorium intybus* L. roots*;* *Elymus repens*(L.) Couldroots; *Fragaria vesca* L. leaves; *Rosa* spp. ^g^leaves; *Urtica dioica* L. leaves*; Vaccinium myrtillus* L. leaves; (1:1:1:1:1:1)	Infusion; 1 table spoon in 200 ml of boiling water	50 ml several times a day	Diabetes	Volynchenko (2003)
6.13	*Arctostaphylos uva-ursi* (L.) Spreng. leaves; *Fragaria vesca* L. leaves; *Vaccinium vitis-idaea* L. leaves; *Rosa* spp*.* ^g^fruits; *Vaccinium myrtillus* L. leaves; *Urtica dioica* L. leaves; (2:2:2:3:4:1)	Infusion; 70 g in 1,000 ml of water, maceration 3–5 h in a dark place	3–4 times a day after eating	Diabetes	Brusenskaya and Kaz’min (2005)
6.14	*Ribes nigrum* L. leaves; *Hypericum* spp*.* ^e^ flowers; *Sambucus nigra* L. flowers; *Urtica dioica* L. leaves; *Juglans regia* L. leaves; *Fragaria vesca* L. leaves; (4:5:4:3:4:3)	Infusion (herbal tea) 1 table spoon in 200 ml (1 glass) of boiling water	100 ml, 2 times a day; before eating	Diabetes	Brusenskaya and Kaz’min (2005)
6.15	*Vaccinium myrtillus* L. leaves; *Urtica dioica* L. leaves; *Phaseolus vulgaris* L. pericarp; *Taraxacum campylodes* G.E.Haglund. roots; *Salvia officinalis* L. leaves; *Galega officinalis* L. aerial part; (5:5:3:1:1:5)	Infusion; 1 table spoon in 500 ml of boiling water, maceration 12 h in thermos	100 ml (warm) 2–3 times a day 30 min before eating	Diabetes	Sinyakov (1992), Sinyakov (1999); Chirkov and Seryi (1993)
6.16	*Hypericum* spp. ^e^ aerial part; *Achillea millefolium* L. aerial part; *Plantago major* L. leaves; *Arctium* spp*.* ^b^ roots; *Centaurium erythraea* Rafn aerial part; *Matricaria chamomilla* L. flowers; (6:2:2:2:1:3)	Infusion; 80 g in 1,000 ml of boiling water, maceration 5–7 h at room temp.	66 ml 15–20 min before eating	Diabetes accompanied by colitis and constipation	Brusenskaya and Kaz’min, (2005)
6.17	*Phaseolus vulgaris* L. pericarp; *Morus* spp.^i^leaves; *Juglans regia* L. leaves; *Acorus calamus* L. root; *Frangula alnus* Mill. bark; *Ribes nigrum* L*.* leaves (20:5:5:5:3:15)	Infusion; 53 g in 1,000 ml of boiling water, maceration 10–12 h at room temp.	3 table spoons 20–30 min before eating	Diabetes accompanied by colitis and constipation	Brusenskaya and Kaz’min, (2005)
6.18	*Helichrysum (arenarium *(L.) Moench. flowers; *Hypericum* spp. ^e^aerial part; *Polygonum aviculare* L. aerial part; *Rosa spp.* ^g^fruits; *Vaccinium myrtillus* L. leaves; *Zea mays* L. corn silk; (2:2:3:2:2:2)	Infusion; 65 g in 1,000 ml of boiling water, maceration 10–12 h at room tempature	50–70 ml (warm) 3–4 times a day before eating	Diabetes accompanied by liver and gallbladder diseases	Brusenskaya and Kaz’min (2005); Bogdanova and Bashkirova (2010)
6.19	*Vaccinium myrtillus* L. leaves; *Helichrysum (arenarium *(L.) Moench flowers; *Zea mays* L. corn silk; *Polygonum aviculare* L. aerial part; *Hypericum* spp*.* ^e^aerial part; *Phaseolus vulgaris* L. pericarp; (4:1:2:1:2:2)	Infusion; 60 g in 1,000 ml of boiling water, maceration 10–12 h at room tempature	50–70 ml (warm) 3–4 times a day before eating	Diabetes accompanied by liver and gallbladder diseases	Brusenskaya and Kaz’min, (2005)
6.20	*Achillea millefolium* L. aerial part; *Matricaria chamomilla* L. flowers; *Hypericum* spp*.* ^e^aerial part; *Mentha × piperita* L. leaves; *Quercus* spp.^h^bark; *Tanacetum vulgare* L. flowers; (30:8:20:5:15:8)	Infusion; 86 g in 1,000 ml of boiling water, maceration 3–4 h at room tempature	50 ml 3–4 times a day before eating for 7–10 days	Diabetes with frequent diarrhea	Brusenskaya and Kaz’min, (2005)
6.21	*Betula pendula* Roth*.* leaves; *Foeniculum vulgare* Mill. fruits; *Frangula alnus* Mill. bark; *Glycyrrhiza glabra* L*.* roots; *Sambucus nigra* L. flowers; *Viola tricolor* L. aerial part; (1:1:1:1:1:1)	Decoction; 1 table spoon in 200 ml of boiling water	66 ml 3 times a day	Skin rash, metabolic disorder	Chirkov and Seryi, (1993)
6.22	*Arctium lappa* L*.* roots; *Elymus repens (*L.*)* Gould rhizomes; *Foeniculum vulgare* Mill. fruits; *Frangula alnus* Mill. bark; *Glycyrrhiza glabra* L*.* roots; *Taraxacum campylodes* G.E.Haglund. roots; (1:1:1:1:1:1)	Decoction; 1 table spoon in 200 ml of boiling water	200 ml (warm) in the morning before first eating	Metabolism improving	Chirkov and Seryi, (1993)
6.23	*Arctostaphylos uva-ursi* (L.) Spreng. leaves; *Frangula alnus* Mill. bark; *Herniaria (glabra)* L. aerial part; *Ononis spinosa* L. roots; *Saponaria officinalis* L. roots; *Solanum dulcamara* L. aerial part; (1:1:1:1:1:1)	Decoction; 1 table spoon in 200 ml of boiling water	200–400 ml in the morning before first eating	Metabolism improving and diuretics	Chirkov and Seryi, (1993)
6.24	"Normavit" *Saccharina latissima* (L.) C.E.Lane, C.Mayes, Druehl & G.W.Saunders thallus; *Rosa* spp*.* ^g^fruits; *Vaccinium vitis-idaea* L. leaves; *Leonurus* spp ^d^aerial part; *Bidens (tripartita* L.*)* aerial part; *Frangula alnus* Mill. bark; (4:1:1:1:1:1)	Decoction; 10 g in 130 ml of boiling water	50–100 ml 3 times a day before eating for 20–30 days	Metabolism improving	Samylina et al. (2010)
**7 plants**					
7.1	*Betula* spp*.* ^c^leaves; *Filipendula* *ulmaria*(L.) Maxim. aerial part; *Fragaria vesca* L. leaves; *Hypericum* spp. ^e^aerial part; *Melissa officinalis* L*.* aerial part; *Prunus spinosa* L. flowers; *Rosa* spp. ^g^fruits; (1:1:1:1:1:1:1)	Infusion; 10 g in 300 ml of boiling water, boil 5 min, maceration 2 h in thermos	100 ml 3–4 times a day 30 min before eating	Obesity	Vinogradova et al. (2001); Turishchev (2000), Turishchev (2005)
7.2	*Achillea millefolium* L. aerial part; *Matricaria chamomilla* L. flowers; *Mentha × piperita* L*.* leaves; *Ribes nigrum* L. leaves; *Sorbus aucuparia* L. fruits; *Vaccinium myrtillus* L. shoots; *Vaccinium vitis-idaea* L. leaves; (1:1:1:1:1:1:1)	Infusion; 10 g in 300 ml of boiling water, boil 5 min, maceration 3 h in thermos	100 ml 3–4 times a day 15 min before eating	Obesity	Vinogradova et al. (2001); Turishchev (2000), Turishchev (2005)
7.3	*Tilia cordata* Mill. flowers; *Rosa majalis* Herrm. fruits; *Betula* spр. ^c^leaves; *Origanum vulgare* L aerial part; *Hypericum perforatum* L. aerial part; *Calendula officinalis* L. flowers; *Ribes nigrum* L. leaves; (3:3:1:1:1:2:2)	Infusion; 2 table spoons in 2 400 ml of boiling water, maceration 8 h in thermos	100 ml 3 times a day	Obesity	Kukes (1999); Bubenchikova et al. (2003)
7.4	*Zea mays* L. corn silk; *Frangula alnus* Mill*.* bark; *Cichorium intybus* L. roots; *Taraxacum campylodes* G.E.Haglund. roots; *Mentha × piperita* L*.* leaves *Petroselinum crispum* (Mill.) Fuss fruits; *Foeniculum vulgare* Mill. fruits; (5:3:3:2:1:1:1)	Infusion; 2 table spoons in 500 ml of boiling water	100 ml 4 times a day	Obesity	Lager (1991), Lager (2002)
7.5	"Arfazetin" *Vaccinium myrtillus* L. shoots; *Phaseolus vulgaris* L. pericarp; *Aralia elata* (Miq.) Seem roots; (or *Oplopanax elatus* (Nakai) Nakai roots and rhizomes); *Rosa* spp. ^g^fruits; *Equisetum arvense* L. aerial part; *Hypericum perforatum* L. aerial part; *Matricaria chamomilla* L. flowers; (4:4:2:3:3:2:2)	Infusion; 10 g in 400 ml of water	66 ml 2–3 times a day before eating for 20–30 days	Lowering of blood glucose level, improving of glycogen-forming function of the liver, fortifying, anti-inflammatory	Korotkova et al. (1988); Matkovskaya et al. (1988); Efimov and Shcherbak (1993); Nikultseva (1994); Sokolov (2000); Blinov (2000); Turishchev (2000), Turishchev (2005); Mashkovskii (2002); Dremova et al. (2003); Kiyanova (2005); Nazina (2006); Ryzhenko (2007); Davydovich et al. (2008); Vichkanova et al. (2009); Ruzhenkova (2014); Maznev (2014); Letova (2019); Register Russia (2021)
7.6	*Viburnum opulus* L. berries; *Vaccinium myrtillus* L. fruits; *Galega officinalis* L. aerial part; *Vaccinium vitis-idaea* L*.* fresh berries (fruits); *Hypericum* spp*.* ^e^flowers; *Fragaria vesca* L. leaves; *Arctostaphylos uva-ursi* (L.) Spreng. Leaves; (4:6:5:6:4:3:2)	Infusion (herbal tea); 1 table spoon in 200 ml of boiling water	100 ml 2 times a day before eating	Diabetes	Brusenskaya and Kaz’min, (2005)
7.7	*Vaccinium myrtillus* L. leaves; *Urtica dioica* L. leaves; *Phaseolus vulgaris* L. pericarp; *Taraxacum campylodes* G.E.Haglund. roots; *Fragaria vesca* L. leaves; *Betula pendula* Roth. leaves; *Hypericum perforatum* L. aerial part; (5:2:5:2:2:2:2)	Decoction; 1 table spoon in 600 ml of water	50 ml 6 times a day	Diabetes	Chirkov and Seryi (1993); Kukes (1999); Bubenchikova et al. (2003); Dremova et al. (2003)
7.8	*Avena sativa* L. aerial part; *Fragaria vesca* L. aerial part; *Linum usitatissimum* L. seeds; *Melissa officinalis* L*.* aerial part; *Rosa* spp. ^g^fruits; *Taraxacum campylodes* G.E.Haglund. roots; *Vaccinium vitis-idaea* L. leaves; (1:1:1:1:1:1:1)	Infusion; 10 g in 300 ml of boiling water, 15 min in boil water bath, maceration 1 h in thermos	66 ml 4 times a day 15 min before eating	Diabetes	Vinogradova et al. (2001)
7.9	*Fragaria vesca* L. aerial part; *Galega officinalis* L. aerial part; *Helichrysum arenarium* (L.) Moench flowers; *Laurus nobilis* L. leaves; *Levisticum officinale* W.D.J.Koch roots; *Urtica dioica* L. leaves*; Vaccinium vitis-idaea* L*.* leaves; (1:1:1:1:1:1:1)	Infusion; 10 g in 400 ml of boiling water, 15 min in boil water bath, maceration 2 h in thermos	70–100 ml 3–4 times a day before eating	Diabetes	Vinogradova et al. (2001)
7.10	*Equisetum arvense* L. aerial part; *Polygonum aviculare* L. aerial part; *Fragaria vesca* L. leaves; *Astragalus dasyanthus* Pall. aerial part; *Galega officinalis* L. aerial part; *Arnica montana* L. flowers; *Plantago major* L. leaves; (4:4:4:3:3:1:3)	Infusion; 1 table spoons in 200 ml of boiling water, boil 3–5 min, maceration 10–15 min at room tempature	2 table spoons 3–4 times a day 20–30 min before eating	Diabetes	Korsun et al. (2016)
7.11	*Equisetum arvense* L. aerial part; *Vaccinium myrtillus* L. leaves; *Juglans regia* L. leaves; *Phaseolus vulgaris* L. pericarp; *Fragaria vesca* L*.* leaves; *Matricaria chamomilla* L. flowers; *Cichorium intybus* L*.* leaves; (1:3:3:4:1:1:2)	Infusion; 2 table spoons in 400 ml of boiling water, boil 15 min	66 ml 4 times a day 20–30 min before eating	Diabetes	Lavrenova and Lavrenov (2007)
7.12	*Galega officinalis* L. aerial part; *Phaseolus vulgaris* L. pericarp; *Vaccinium myrtillus* L. leaves; *Equisetum arvense* L. aerial part; *Taraxacum campylodes* G.E.Haglund. roots; *Urtica dioica* L. leaves; *Gnaphalium uliginosum* L*.* aerial part; (2:2:2:1:1:1:1)	Infusion; 30 g in 400 ml of boiling water, boil 10 min, maceration 1 h	150 ml 4 times a day 30 min before eating	Diabetes	Lavrenova and Lavrenov (2007)
7.13	"Arfazetin E" *Vaccinium myrtillus* L. shoots; *Phaseolus vulgaris* L. pericarp; *Eleutherococcus senticosus* (Rupr. & Maxim.) Maxim. roots and rhizomes; *Rosa* spp. ^g^fruits; *Equisetum arvense* L. aerial part; *Hypericum perforatum* L. aerial part; *Matricaria chamomilla L.* flowers (4:4:2:3:3:2:2)	Infusion; 10 g in 400 ml of water	70–100 ml 2–3 times a day before eating for 20–30 days	Mild form of diabetes in combination with diet and exercise. Moderate diabetes in combination with oral hypoglycemic drugs or insulin	Register Russia (2021)
7.14	*Acorus calamus* L*.* roots; *Artemisia absinthium* L. aerial part; *Bidens tripartita* L. aerial part; *Mentha × piperita* L. leaves; *Origanum vulgare* L. aerial part; *Pinus sylvestris* L. buds; *Thymus serpyllum* L. aerial part; (2:3:3:3:3:3:2)	Herbal tea; 6 table spoons in 3,000 ml of boiling water	For external use, baths	Metabolic disorder with skin furuncles	Protasenya and Vasilenko (1992)
**8 plants**					
8.1	*Arctostaphylos uva-ursi* (L.) Spreng. leaves; *Foeniculum vulgare* Mill. fruits; *Lavandula angustifolia* Mill. leaves; *Ononis spinosa* L. roots; *Persicaria hydropiper* L. aerial part; *Rheum palmatum* L. roots; *Rosa majalis* Herrm. fruits; *Senna alexandrina* Mill. leaves; (2:2:2:2:2:1:1:1)	Decoction; 2 table spoons in 500 ml of water	100 ml 4 times a day before eating	Obesity	Chirkov and Seryi (1993)
8.2	*Agrimonia eupatoria*L.aerial part;*Arctostaphylos uva-ursi* (L.) leaves; *Cetraria islandica* L. thallus; *Filipendula ulmaria*(L.) Maxim.aerial part;*Fumaria officinalis*L.aerial part;*Juglans regia* L. leaves; *Morus*nigra L.leaves; *Pinus silvestris* L*.* buds; (1:1:1:1:1:1:1:1)	Infusion; 2 table spoons in 500 ml of boiling water	50 ml 3 times a day before eating	Obesity	Osetrov and Shreter (2001); Korsun and Korsun (2010)
8.3	*Achillea millefolium* L*.* aerial part; *Matricaria chamomilla* L. flowers ; *Elymus repens* (L.) Gould rhizomes; *Equisetum arvense* L. aerial part; *Fucus vesiculosus* L. thallus; *Hypericum perforatum* L. aerial part; *Melissa officinalis* L*.* aerial part; *Taraxacum campylodes* G.E.Haglund. roots*;* (1:1:1:1:2:1:1:1)	Infusion; 1 tea spoon in 200 ml of boiling water	200 ml 2 times a day	Obesity	Osetrov (1993)
8.4	*Achillea millefolium L.,* aerial part; *Anethum graveolens* L. fruits; *Frangula alnus* Mill. bark; *Helichrysum arenarium* (L.) Moench flowers; *Orthosiphon aristatus* (Blume) Miq. shoots; *Rosa* spp. ^g^fruits; *Taraxacum campylodes* G.E.Haglund. roots; *Zea mays* L. corn silk; (1:1:1:1:1:1:1:1)	Infusion; 15 g in 500 ml of cold water, maceration 4 h at room temp., boil 3 min, maceration 1 h in thermos	100 ml 4–5 times a day before eating	Obesity	Vinogradova et al. (2001); Turishchev (2000), Turishchev (2005)
8.5	*Tilia cordata* Mill. flowers; *Rosa* spp*.* ^g^fruits; *Betula spр.* ^c^leaves; *Origanum vulgare* L. aerial part; *Hypericum* spp*.* ^e^aerial part; *Calendula officinalis* L. flowers; *Ribes nigrum* L. leaves; *Gnaphalium uliginosum* L. aerial part; (3:3:1:1:1:1:2:2)	Infusion; 10 g in 500 ml of boiling water, maceration 6–8 h in thermos	125 ml 3–4 times a day before eating	Obesity and diabetes mellitus	Kolesova et al. (1998)
8.6	*Arctium* spp.^b^leaves; *Avena sativa* L. aerial part; *Juniperus communis* L. fruits; *Linum usitatissimum* L. seeds; *Phaseolus vulgaris* L. pericarp; *Taraxacum campylodes* G.E.Haglund. roots; *Urtica dioica* L. leaves; *Vaccinium myrtillus* L. leaves; (1:1:1:1:1:1:1:1)	Infusion; 8 g in 500 ml of boiling water, maceration 6–8 h in thermos	1 table spoon 6 times a day	Lowering of blood glucose level	Podduev (2001)
8.7	*Equisetum arvense* L. aerial part; *Oplopanax* *elatus*(Nakai) Nakairoots and rhizomes; *Taraxacum campylodes* G.E.Haglund. roots; *Rosa* spp*.* ^g^fruits; *Cichorium intybus* L. roots; *Linum usitatissimum* L. seeds; *Hypericum* spp*.* ^e^aerial part; *Tilia cordata* Mill. flowers; (4:1:1:2:3:1:2:1)	Infusion (herbal tea); 1 table spoon in 200 ml of boiling water	100 ml 2 times a day before eating	Diabetes	Brusenskaya and Kaz’min (2005)
8.8	*Arctium* spp*.* ^b^ roots; *Glycyrrhiza glabra* L. roots; *Juglans regia* L. leaves; *Linum usitatissimum* L. seeds; *Rosa* spp.^g^fruits; *Sambucus nigra* L. root; *Vaccinium myrtillus* L. shoots ; *Viburnum opulus* L. shoots; (1:1:1:1:1:1:1:1)	Infusion; 10 g in 400 ml of boiling water, 15 min in boil water bath, maceration 2 h in thermos	70–100 ml 3–4 times a day before eating	Diabetes	Vinogradova et al. (2001)
8.9	*Matricaria chamomilla* L. flowers; *Viola tricolor* L. aerial part; *Equisetum arvense* L. aerial part; *Achillea millefolium* L. aerial part; *Calendula officinalis *L. flowers; *Quercus* spp. ^h^bark; *Gnaphalium uliginosum* L*.* aerial part; *Melilotus officinalis* (L.) Pall*.*aerial part; (2:1:1:2:1:2:1:1)	Infusion; 1 tea spoon in 200 ml of boiling water, 15 min in boil water bath, maceration 45 min in thermos	100 ml 2 times a day after eating	Diabetes	Korsun et al. (2016)
8.10	*Taraxacum campylodes* G.E.Haglund. leaves; *Vaccinium vitis-idaea* L. leaves; *Galega officinalis* L. aerial part; *Polygonum aviculare* L. aerial part; *Ribes nigrum* L. leaves; *Phaseolus vulgaris* L. pericarp; *Cichorium intybus* L*.* leaves; *Rosa* spp.^g^fruits; (1:4:4:1:2:3:2:1)	Infusion; 2 table spoons in 400 ml of boiling water, boil 8 min, maceration 2 h	100 ml 4 times a day 20–30 min before eating	Diabetes	Lavrenova and Lavrenov (2007)
**9 plants**					
9.1	*Matricaria chamomilla* L. flowers; *Equisetum arvense* L. aerial part; *Filipendula* *ulmaria*(L.) Maxim. aerial part; *Foeniculum vulgare* Mill. fruits; *Glycyrrhiza glabra* L. roots; *Hypericum* spp*.* ^e^aerial part; *Mentha × piperita* L*.* aerial part; *Sambucus nigra* L. root; *Stachys officinalis* (L.) Trevis*.* aerial part; (1:1:1:1:1:1:1:1:1)	Infusion; 15 g in 500 ml of boiling water, 15 min in boil water bath, maceration 2 h in thermos	100 ml 4–5 times a day 20 min before eating	Obesity	Vinogradova et al. (2001)
9.2	*Betula* spp*.* ^c^leaves; *Foeniculum vulgare* Mill. fruits; *Fragaria vesca* L. aerial part; *Mentha × piperita* L*.* aerial part; *Petroselinum crispum* (Mill.) Fuss aerial part; *Phaeophyceae* (*Cystoseira barbata* (Stackh.) C.Agardh) thallus; *Polygonum aviculare* L. aerial part; *Rosa* spp.^g^fruits; *Urtica dioica L.* leaves; (1:1:1:1:1:1:1:1:1)	Infusion; 10 g in 300 ml of boiling water, boil 5 min, maceration 2 h in thermos	100 ml 3–4 times a day 30 min before eating	Obesity	Vinogradova et al. (2001)
9.3	*Bidens tripartita* L. aerial part; *Matricaria chamomilla* L. flowers; *Equisetum arvense* L. aerial part; *Hypericum perforatum* L*.* aerial part; *Inula helenium* L. roots; *Mentha × piperita* L*.* leaves; *Oplopanax* *elatus*(Nakai) Nakairoots and rhizomes;*Rosa majalis* Herrm. fruits; *Vaccinium myrtillus* L. leaves; (1:1:1:1:1:1:1:2:1)	Infusion; 10 g in 400 ml of water	66 ml 3 times a day	Lowering of blood glucose level	Sokolov and Zamotaiev (1984); Lisitsyn and Molodozhnikova (1989); Lager (1991), Lager (2002); Efimov and Shcherbak (1993); Nikultseva (1994); Tarasenko et al. (1998); Sinyakov (1999); Sokolov (2000); Dontsov and Dontsov (2000); Blinov (2000); Sokolov (2000); Dremova et al. (2003); Smolianskii and Lifliandskii (2004); Davydovich et al. (2008); Bogdanova and Bashkirova (2010); Ruzhenkova (2014); Maznev (2014)
9.4	*Bidens tripartita* L. aerial part; *Matricaria chamomilla* L. flowers; *Equisetum arvense* L. aerial part; *Fragaria vesca* L*.* roots; *Hypericum perforatum* L. aerial part; *Inula helenium* L*.* roots; *Mentha × piperita* L. leaves; *Rosa majalis* Herrm. fruits; *Vaccinium myrtillus* L. leaves; (1:1:1:4:1:1:1:1:2)	Infusion; 1 table spoon in 200 ml of boiling water	66 ml 3 times a day before eating	Diabetes	Podduev (2001)
9.5	*Crataegus sanguinea* Pall*.* fruits; *Rosa majalis* Herrm. fruits; *Urtica dioica* L. leaves; *Leonurus quinquelobatus* Gilib. aerial part; *Linum usitatissimum* L*.* seeds; *Mentha × piperita* L. leaves; *Asparagus officinalis* L. rhizomes and aerial part; *Thymus serpyllum* L*.* aerial part; *Vaccinium myrtillus* L. leaves; (3:3:3:5:2:1:4:4:7)	Infusion; 2–3 table spoons in 500 ml of boiling water, maceration 12 h in thermos	66 ml (warm) 3 times a day 20–30 min before eating	Diabetes	Ladynina and Morozova (1987), Ladynina and Morozova (1990); Blinov (2000); Sinyakov (1999); Nazina (2006); Davydovich et al. (2008); Bogdanova and Bashkirova (2010)
9.6	Carex arenaria L. rhizomes; *Cyanus* *segetum*Hillaerial part; *Galega officinalis* L. aerial part; *Phaseolus vulgaris* L. pericarp; *Pimpinella saxifraga*L. roots;*Salvia officinalis* L. leaves; *Sambucus nigra* L. flowers; *Taraxacum campylodes* G.E.Haglund. roots; *Vaccinium myrtillus* L. leaves; (1:1:1:1:1:1:1:1:2)	Infusion; 2 table spoons in 500 ml of boiling water, 15 min in boil water bath, maceration 30–40 min	66 ml 3 times a day 30 min before eating	Diabetes	Sinyakov (1999)
9.7	*Arctium lappa* L. roots; *Capsella bursa-pastoris* (L.) Medik. aerial part; *Hypericum perforatum* L. aerial part; *Juglans regia* L. leaves; *Mentha × piperita* L. leaves; *Rosa majalis* Herrm. fruits; *Vaccinium myrtillus* L. leaves; *Vaccinium vitis-idaea* L. leaves; *Zea mays* L. corn silk; (6:3:3:4:1:4:4:5:5)	Infusion; 2 table spoons in 500 ml of boiling water, maceration 12 h in thermos	100 ml 3–4 times a day 30 min before eating	Diabetes	Sinyakov (1999)
9.8	*Crataegus* spp*.* fruits; *Fragaria vesca* L*.* leaves; *Hypericum spp.* ^e^aerial part; *Linum usitatissimum* L. seeds; *Phaseolus vulgaris* L. pericarp; *Plantago major* L. leaves; *Ribes nigrum* L. leaves; *Rosa* spp. fruits; *Vaccinium myrtillus* L. leaves; (1:1:1:1:1:1:1:1:2)	Infusion; 3 table spoons in 500 ml of boiling water, maceration 12 h in thermos	140 ml (warm) 3 times a day 30 min before eating	Diabetes	Podduev (2001)
**10 plants**					
10.1	*Anethum graveolens* L. fruits; *Matricaria chamomilla* L. flowers; *Frangula alnus* Mill. bark; *Hypericum* spp*.* ^e^aerial part; *Juniperus communis* L. fruits; *Prunus spinosa* L. flowers; *Rosa* spp.^g^fruits; *Taraxacum campylodes* G.E.Haglund*.* roots; *Urtica dioica* L. leaves*; Zea mays* L. corn silk; (1:1:1:1:1:1:1:1:1:1)	Infusion; 10 g in 300 ml of boiling water, maceration 3 h in thermos	100 ml 3–4 times a day 15 min before eating	Obesity	Vinogradova et al. (2001)
10.2	*Betula* spp*.* ^c^leaves; *Cichorium intybus* L. roots; *Frangula alnus* Mill*.* bark; *Glycyrrhiza glabra* L. roots; *Mentha × piperita* L*.* aerial part; *Morus* spp.^i^ leaves; *Orthosiphon aristatus* (Blume) Miq. shoots; *Petroselinum crispum* (Mill.) Fuss roots; *Taraxacum campylodes* G.E.Haglund. roots; *Zea mays* L. corn silk; (1:1:1:1:1:1:1:1:1:1)	Infusion; 15 g in 500 ml of boiling water, 15 min in boil water bath, maceration 2 h in thermos	100 ml 4–5 times a day 20 min before eating	Obesity	Vinogradova et al. (2001)
10.3	*Vaccinium vitis-idaea* L*.* leaves; *Zea mays* L. corn silk; *Syringa vulgaris* L. buds; *Arctium lappa* L*.* roots; *Mentha × piperita* L. leaves; *Juglans regia* L*.* leaves; *Hypericum perforatum* L*.* aerial part; *Gnaphalium uliginosum* L*.* aerial part; *Vaccinium myrtillus* L. leaves; *Rosa majalis* Herrm*.* fruits; (4:4:2:5:2:3:2:2:3:1)	Infusion; 2–3 table spoons in 500 ml of boiling water, maceration 12 h in thermos	66 ml (warm) 3 times a day,20–30 min before eating	Diabetes	Ladynina and Morozova (1987), Ladynina and Morozova (1990); Blinov, 2000; Ladynina (2005); Nazina, (2006)
10.4	*Achillea millefolium L.* aerial part; *Arctium* spp*.* ^b^ roots; *Elymus* *repens*(L.) Gouldrhizomes; *Fragaria vesca* L. aerial part; *Galega officinalis* L. aerial part; *Laurus nobilis* L. leaves; *Phaeophyceae* (*Cystoseira barbata* (Stackh.) C.Agardh) thallus; *Rosa* spp.^g^fruits; *Trifolium pratense* L. flowers; *Vaccinium myrtillus* L. leaves; (1:1:1:1:1:1:1:1:1:1)	Infusion; 12 g in 350 ml of boiling water, 10 min in boil water bath, maceration 2 h in thermos.	100 ml (warm) 3 times a day10 min before eating	Diabetes	Vinogradova et al. (2001)
10.5	*Arctium lappa* L. roots; *Artemisia absinthium* L. aerial part; *Bidens tripartita* L. aerial part; *Calendula officinalis *L. flowers; *Matricaria chamomilla* L. flowers; *Equisetum arvense* L. aerial part; *Gnaphalium uliginosum* L. aerial part; *Hypericum perforatum* L. aerial part; *Inula helenium* L. roots; *Salvia officinalis* L. aerial part; (1:1:1:1:1:1:1:1:1:1)	Decoction; 1–2 table spoons in 200 ml of water	70–100 ml 3 times a day 30 min before eating	Diabetes accompanied by chronic pancreatitis	Chirkov and Seryi, (1993)
**11 plants**					
11.1	*Achillea millefolium* L. aerial part; *Arctium* spp.^b^roots; *Cichorium intybus* L. roots; *Elymus* *repens*(L.) Gouldroot; *Inula helenium* L. roots; *Phaseolus vulgaris* L. pericarp; *Polygonum aviculare* L. aerial part; *Ribes nigrum L.* leaves; *Taraxacum campylodes* G.E.Haglund. roots; *Tilia cordata* Mill. flowers; *Vaccinium myrtillus* L. leaves; (1:2:1:2:1:2:2:1:2:2:2)	Decoction; 10 g in 500 ml of water, 2 h in boil water bath	100–150 ml 3–4 times a day before eating	Insulin-dependent diabetes	Kolesova et al. (1998)
11.2	*Betula pubescens* Ehrh*.* leaves; *Crataegus sanguinea* Pall. fruits; *Orthosiphon aristatus* (Blume) Miq. leaves; *Rosa majalis* Herrm. fruits; *Mentha × piperita* L. leaves; *Veronica officinalis* L. aerial part; *Centaurium erythraea* Rafn aerial part; *Arctium lappa* L. roots; *Leonurus quinquelobatus* Gilib. aerial part; *Glycyrrhiza glabra* L. roots; *Cichorium intybus* L. roots (2:3:2:2:2:1:5:5:3:2:4)	Infusion; 2–3 table spoons in 500 ml of boiling water, maceration 12 h in thermos	66 ml (warm) 3 times a day 20–30 min before eating	Diabetes	Ladynina and Morozova (1987), Ladynina and Morozova (1990); Blinov (2000); Dremova et al. (2003); Ladynina (2005); Nazina (2006); Bogdanova and Bashkirova (2010); Maznev (2014)
11.3	*Vaccinium myrtillus* L. leaves; *Phaseolus vulgaris* L. pericarp; *Crataegus sanguinea* Pall. Fruits; *Hypericum perforatum* L. aerial part; *Plantago major* L. leaves; *Ribes nigrum* L. leaves; *Rosa majalis* Herrm*.* fruits; *Linum usitatissimum* L. seeds; *Mentha × piperita* L*.* leaves; *Fragaria vesca* L*.* leaves *Sambucus nigra* L. flowers; (4:3:2:2:2:2:1:1:1:1:1)	Infusion; 3 table spoons in 500 ml of boiling water, maceration 12 h in thermos	130–140 ml (warm) 3 times a day 20–30 min before eating	Diabetes	Sinyakov (1992); Sinyakov (1999); Tarasenko et al. (1998); Davydovich et al. (2008)
11.4	*Artemisia absinthium* L. aerial part; *Avena sativa* L. aerial part; *Urtica dioica* L. leaves; *Arctium* spp*.* ^b^roots; *Inula helenium* L. roots; *Alchemilla xanthochlora* Rothm. aerial part; *Taraxacum campylodes* G.E.Haglund. leaves; *Vaccinium myrtillus* L. leaves; *Gnaphalium uliginosum* L*.* aerial part; *Sambucus nigra* L. flowers; *Linum usitatissimum* L. seeds; (4:4:4:2:1:2:1:2:4:4:2)	Infusion; 2 table spoons in 500 ml of boiling water, maceration 12 h at room temp.	100 ml 3 times a day 15 min before eating.	Diabetes	Rendiuk (2006)
11.5	*Alchemilla xanthochlora* Rothm. aerial part; *Centaurium erythraea* Rafn aerial part; *Elymus* *repens*(L.) Couldrhizomes; *Gnaphalium uliginosum* L*.* aerial part; *Juniperus communis* L. fruits; *Mentha × piperita* L*.* aerial part; *Rumex confertus* Willd. roots; *Sorbus aucuparia* L. fruits; *Syringa vulgaris* L. buds; *Taraxacum campylodes* G.E.Haglund. roots; *Trifolium pratense* L. flowers; (1:1:1:1:1:1:1:1:1:1:1)	Infusion; 12 g in 350 ml of boiling water, 10 min in boil water bath, maceration 2 h in thermos	100 ml (warm) 3 times a day 10 min before eating	Diabetes	Vinogradova et al. (2001)
11.6	*Arctium* spp*.* ^b^ roots; *Avena sativa* L. aerial part; *Betula* spp*.* ^c^leaves; *Galega officinalis* L. aerial part; *Glycyrrhiza glabra* L. roots; *Hypericum* spp*.* ^e^aerial part; *Juglans regia* L. leaves; *Juniperus communis* L. fruits; *Laurus nobilis* L. leaves; *Vaccinium myrtillus* L. leaves ; *Vaccinium vitis-idaea* L*.* leaves; (1:1:1:1:1:1:1:1:1:1:1)	Infusion; 10 g in 400 ml of cold water, maceration 4 h, boil 3 min, maceration 2 h in thermos	100 ml 3 times a day before eating	Diabetes	Vinogradova et al. (2001)
11.7	*Betula* spp*.* ^c^leaves; *Cyanus segetum* Hill flowers; *Foeniculum vulgare* Mill. fruits; *Laurus nobilis* L. leaves; *Linum usitatissimum* L. seeds; *Ononis spinosa* L. roots; *Petroselinum crispum* (Mill.) Fuss roots; *Phaseolus vulgaris* L. pericarp; *Taraxacum campylodes* G.E.Haglund. roots; *Urtica dioica* L. roots; *Viburnum opulus* L. flowers; (1:1:1:1:1:1:1:1:1:1:1)	Infusion; 12 g in 400 ml of boiling water, 15 min in boil water bath, maceration 1.5 h in thermos	70–100 ml 3–4 times a day 10 min before eating	Diabetes	Vinogradova et al. (2001)
11.8	*Arctium lappa* L. roots; *Centaurium erythraea* Rafn aerial part; *Cichorium intybus* L. roots; *Crataegus sanguinea* Pall. fruits; *Rosa majalis* Herrm. fruits; *Glycyrrhiza glabra* L. roots; *Leonurus quinquelobatus* Gilib. aerial part; *Orthosiphon aristatus* (Blume) Miq. leaves; *Mentha × piperita* L. leaves; *Betula nigra* L. leaves; *Veronica officinalis* L. aerial part; (3:3:2:2:2:2:2:1:1:1:1)	Infusion; 2–3 table spoons in 500 ml of boiling water, maceration 12 h in thermos	130 ml (warm) 3 times a day 30 min before eating	Diabetes	Sinyakov (1999)
**12 plants**					
12.1	*Crataegus sanguinea* Pall. fruits; *Mentha × piperita L.* leaves; *Rosa majalis* Herrm. fruits; *Sambucus nigra* L*.* flowers; *Hypericum perforatum* L*.* aerial part; *Fragaria vesca* L*.* leaves; *Plantago major* L*.* leaves; *Ribes nigrum* L. leaves; *Saccharina latissima* (L.) C.E.Lane, C.Mayes, Druehl & G.W.Saunders thallus; *Linum usitatissimum* L*.* seeds; *Phaseolus vulgaris* L. pericarp; *Vaccinium myrtillus* L. leaves; (3:2:2:2:3:1:3:3:2:2:5:7)	Infusion; 2–3 table spoons in 500 ml of boiling water, maceration 12 h in thermos	66 ml (warm) 3 times a day 20–30 min before eating	Diabetes	Ladynina and Morozova (1987), Ladynina and Morozova (1990); Lisitsyn and Molodozhnikova (1989); Lager (1991); Lager (2002); Efimov and Shcherbak (1993); Sinyakov (1999); Blinov (2000); Ladynina (2005); Nazina (2006); Bogdanova and Bashkirova (2010);
12.2	"Myrphasinum" *Vaccinium myrtillus* L. shoots; *Phaseolus vulgaris* L. pericarp; *Rosa* spp.^g^fruits; *Urtica dioica* L. leaves; *Plantago major* L. leaves; *Matricaria chamomilla* L. flowers; *Calendula officinalis *L. flowers; *Leonurus* spp ^d^ aerial part; *Hypericum* spp.^e^ aerial part; *Achillea millefolium* L. aerial part; *Glycyrrhiza glabra* L*.* roots; *Inula helenium* L. roots; (2:2:1:1:1:1:1:1:1:1:1:1)	Infusion; 10 g in 400 ml of boiling water	100 ml 2–3 times a day 30 min before eating for 20–30 days	Mild forms of diabetes	Turishchev (2000); Dremova et al. (2003); Belodubrovskaya et al. (2004); Turishchev (2005); Davydovich et al. (2008); Ruzhenkova (2014)
**13 plants**					
13.1	*Avena sativa* L. aerial part; *Capsella bursa-pastoris* (L.) Medik. aerial part; *Cyanus segetum* Hill flowers; *Frangula alnus* Mill. bark; *Laurus nobilis* L. leaves; *Petroselinum crispum* (Mill.) Fuss aerial part; *Phaseolus vulgaris* L. pericarp; *Pimpinella anisum* L. fruits; *Rumex confertus* Willd. roots; *Syringa vulgaris* L. buds; *Taraxacum campylodes* G.E.Haglund. roots; *Tilia cordata* Mill. flowers; *Vaccinium myrtillus* L. leaves ; (1:1:1:1:1:1:1:1:1:1:1:1:1)	Infusion; 12 g in 350 ml of boiling water, 10 min in boil water bath, maceration 2 h in thermos	100 ml (warm) 3 times a day 10 min before eating	Diabetes	Vinogradova et al. (2001)
13.2	*Alchemilla xanthochlora* Rothm. leaves; *Arctostaphylos uva-ursi* (L.) Spreng. leaves; *Centaurium erythraea* Rafn aerial part; *Dioscorea spp.* root; *Helichrysum arenarium* (L.) Moench flowers; *Juniperus communis* L. fruits; *Mentha × piperita* L*.* aerial part; *Morus alba* L. leaves; *Plantago major* L. leaves; *Ribes nigrum* L. leaves; *Saccharina latissima* (L.) C.E.Lane, C.Mayes, Druehl & G.W.Saunders thallus; *Vaccinium vitis-idaea* L*.* leaves; *Veronica officinalis* L. aerial part; (1:1:1:1:1:1:1:1:1:1:1:1:1)	Infusion; 12 g in 350 ml of boiling water, 10 min in boil water bath, maceration 2 h in thermos	100 ml (warm) 3 times a day 10 min before eating	Diabetes	Vinogradova et al. (2001)
13.3	*Juglans regia* L. leaves; *Laurus nobilis* L. leaves; *Morus* spp.^i^leaves; *Phaseolus vulgaris* L. pericarp; *Vaccinium myrtillus* L. shoots; *Avena sativa* L. aerial part; *Fragaria vesca* L. leaves; *Hypericum* spp*.* ^e^aerial part; *Linum usitatissimum* L. seeds; *Plantago major* L. leaves; *Taraxacum campylodes* G.E.Haglund. roots; *Urtica dioica* L. leaves*; Veronica officinalis* L. aerial part; (3:3:3:3:3:1:1:1:1:1:1:1:1)	Infusion; 15 g in 400 ml of boiling water, 15 min in boil water bath, maceration 2 h in thermos	100 ml 4 times a day, 15 min before eating	Diabetes	Vinogradova et al. (2001)
**14 plants**					
14.1	*Achillea millefolium* L. aerial part; *Betula* spp*.* ^c^leaves; *Matricaria chamomilla* L. flowers; *Equisetum arvense* L. aerial part; *Foeniculum vulgare* Mill. fruits; *Frangula alnus* Mill. bark; *Glycyrrhiza glabra* L. roots; *Hypericum* spp*.* ^e^aerial part; *Juniperus communis* L. fruits; *Linum usitatissimum* L. seeds; *Melissa officinalis* L*.* aerial part; *Petroselinum crispum* (Mill.) Fuss roots; *Ribes nigrum* L. leaves; *Urtica dioica* L. leaves*;* (1:1:1:1:1:1:1:1:1:1:1:1:1:1)	Infusion; 15 g in 500 ml of boiling water, 15 min in boil water bath, maceration 2 h in thermos	100 ml 4–5 times a day 20 min before eating	Obesity	Vinogradova et al. (2001)
14.2	*Anethum graveolens* L. fruits; *Crataegus* spp*.* ^f^fruits; *Fragaria vesca* L. leaves; *Helichrysum arenarium* (L.) Moench flowers; *Laurus nobilis* L. leaves; *Linum usitatissimum* L. seeds; *Mentha × piperita* L*.* aerial part; *Orthosiphon aristatus* (Blume) Miq. shoots; *Polygonum aviculare* L. aerial part; *Prunus spinosa* L. flowers; *Rheum palmatum* L. roots; *Rosa* spp.^g^fruits; *Salvia officinalis* L. leaves; *Sorbus aucuparia* L. fruits; (1:1:1:1:1:1:1:1:1:1:1:1:1:1)	Infusion; 10 g in 500 ml of cold water, maceration 4 h at room temp., boil 3 min, maceration 1 h in thermos	100 ml 4–5 times a day before eating	Obesity	Vinogradova et al. (2001)
14.3	*Juglans regia* L. leaves; *Plantago major* L. seeds; *Syringa vulgaris* L. buds; *Phaseolus vulgaris* L. pericarp; *Vaccinium myrtillus* L. shoots; *Arctium* spp*.* ^b^roots; *Taraxacum campylodes* G.E.Haglund. roots; *Ribes nigrum* L. leaves; *Helichrysum (arenarium* (L.) Moench flowers; *Betula* spp*.* ^c^leaves; *Urtica dioica* L. leaves*; Hypericum* spp*.* ^e^aerial part; *Equisetum arvense* L. aerial part; *Fragaria vesca* L. aerial part; (3:1:1:4:4:4:4:2:2:2:2:2:2:2)	Infusion; 15 g in 400 ml of boiling water, boil 3 min, maceration 3 h in thermos	100 ml 4 times a day 15 min before eating	Diabetes	Vinogradova et al. (2001)
14.4	*Laurus nobilis* L. leaves; *Vaccinium myrtillus* L. shoots; *Phaseolus vulgaris* L. pericarp; *Galega officinalis* L. aerial part; *Elymus* *repens*(L.) Couldrhizomes; *Centaurium erythraea* Rafn aerial part; *Melissa officinalis* L*.* aerial part; *Rosa* spp.^g^fruits; *Glycyrrhiza glabra* L. roots; *Vaccinium vitis-idaea* L*.* leaves; *Betula* spp*.* ^c^leaves; *Linum usitatissimum* L. seeds; *Avena sativa* L. aerial part; *Trifolium pratense* L. flowers; (4:4:4:4:2:2:2:2:1:1:1:1:1:1)	Infusion; 15 g in 400 ml of boiling water, 15 min in boik water bath, maceration 2 h in thermos.	100 ml 4 times a day 15 min before eating	Diabetes	Vinogradova et al. (2001)
**15 plants**					
15.1	*Arctium* spp*.* ^b^roots; *Galega officinalis* L. aerial part; *Laurus nobilis* L. leaves; *Phaseolus vulgaris* L. pericarp; *Vaccinium myrtillus* L. shoots ; *Vaccinium vitis-idaea* L*.* leaves; *Betula* spp.^c^leaves; *Centaurium erythraea* Rafn aerial part; *Linum usitatissimum* L. seeds; *Rosa* spp.^g^fruits; *Syringa vulgaris* L. buds; *Melissa officinalis* L*.* aerial part; *Sorbus aucuparia* L. fruits *Trifolium pratense* L. flowers; *Viburnum opulus* L. flowers; (3:3:3:3:3:3:2:2:2:2:2:1:1:1:1)	Infusion; 15 g in 400 ml of boiling water, 15 min in boil water bath, maceration 2 h in thermos.	100 ml 4 times a day 15 min before eating	Diabetes	Vinogradova et al. (2001)

a Code include number of plants and species number (for example 2.1 is mean 2 plants, species 1).

b Arctium *spp.* = Arctium lappa *L.,* A. tomentosum *Mill.,* A. minus *(Mill.)* Bernh.

c Betula *spp.* = Betula pendula *Roth.,* B. pubescens *Ehrh*.

d Leonurus *spp.* = Leonurus quinquelobatus *Gilib.,* L. cardiaca *L*.

e Hypericum *spp.* = Hypericum perforatum *L.,* H. maculatum *Crantz.*

f Crataegus *spp.* = Crataegus laevigata *(Poir.) DC.* (C. oxyacantha sensu *Pojark.*), C. korolkovii *L., Henry,* C.chlorocarpa *Lenne* et C. koch (C. altaica (Lond.) Lange), C. dahurica Koehne ex Schneid., C. monogina *Jacq*., C.alemanniensis *Cin.,* C. pentagyna *Waldst*. et *Kit*., C. orientobaltica *Cin.,* C. curvisepala *Lindm.,* C. x curonica *Cin.,*C. x dunensis *Cin*.

g Rosa *spp.* = Rosa majalis *Herrm.* (R. cinnamomea *L*.); R. acicularis *Lindl.*; R. davurica *Pall.*; R. beggeriana *Schrenk.*; R. fedtschenkoana *Regel.*; R. rugosa *Thunb.* *et al*.

h Quercus *spp.* = Quercus. robur *L. and* Q. petraea *(Mattuschka) Liebl*.

i Morus spp. = Morus alba L., M. nigra L.

## Results and Discussion

For centuries, medicinal plants have been used in Russia for the management of diabetes and related disorders (Zmeev, 1896; Turova and Sapozhnikova, 1989; Minaeva, 1991; Protasenya and Vasilenko, 1992; Nazina, 2007; Chekina et al., 2010; Korsun et al., 2016; Povydysh et al., 2018). The focus of the current paper is on medical species used for the therapy of diabetes and related diseases in traditional and officinal Russian medicine.

### The Characteristics of the Composition of Medical Species, Their Preparation and Posology

We collected information about the composition, specificity of preparation, and posology of 227 species (Table 1). The majority of the species (148) are suggested for the treatment of diabetes; 37 species are recommended for the management of obesity, and eight species are indicated for lowering blood glucose. Several species are recommended for specific cases when diabetes is accompanied by impotence in men (5 species), diabetes accompanied by liver and gallbladder diseases (3 species), diabetes accompanied by gastritis (2 species), etc. Altogether, 158 plants are mentioned in medical species, among which 96 medicinal plants are monographed in the State Pharmacopoeia of the Russian Federation and used in officinal medicine (Shikov et al., 2021). A significant proportion of the plants used in medical species are native to Russian flora.

**FIGURE 1:**
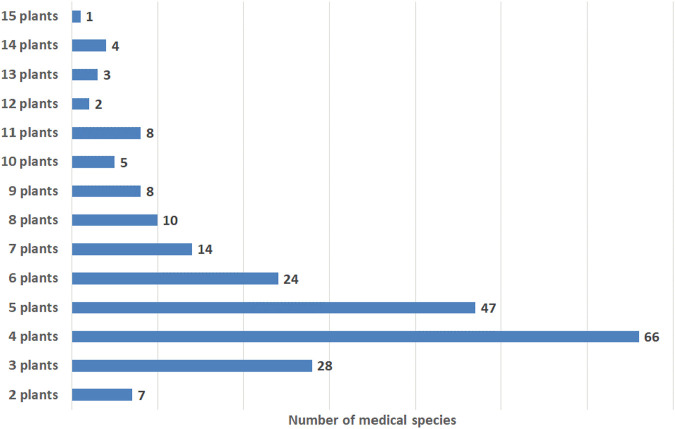
Number of medical species according to the number of plants in the formulation.

Medical species represent mixtures of 2–15 plants. The most frequently mentioned in the literature are medical species comprising mixtures of four plants (66 species), followed by mixtures of five plants (47 species), three plants (28 species), and six plants (24 species) (Figure 1). According to some experts’ opinions, the industrial-scale production of polyherbal mixtures with more than 10 medicinal plants is not rational (Kiseleva and Chauzova, 1999). Indeed, only 18 species among the 227 described contain over 10 plants (Figure 1). However, the numbers of plants in polyherbal mixtures in Ayurveda (Parasuraman et al., 2014), Kampo (Arai et al., 2020), and traditional Chinese medicine (Xutian et al., 2014) are not limited to 10. On the other hand, the species “Myrphasinum”, approved as officinal medicine in Russia, includes 12 plants (Table 1). Although the idea of combining so many plants in one mixture is part of traditional medicine, the quality control of medical species becomes more complicated with each additional component due to challenges related to the specificity of each plant.

Medical species are prepared predominantly in form of infusions or decoctions. Infusions are common for soft plant parts such as aerial parts, leaves, and flowers. Decoctions are preferred for more hard barks, fruits, and roots. The recommended single doses vary from a tablespoon up to 200 ml and depend on the pharmacological activity of the plants in the mixture.

### The Plants Most Frequently Used in Medical Species

The top 10 plants in medical species used for the therapy of diabetes and related disorders (Table 1) include *Vaccinium myrtillus* L. (leaves in 97, shoots in 11, and fruits in 5 species), *Phaseolus vulgaris* L. (pericarp in 65 species), *Taraxacum campylodes* G.E. Haglund. (syn. *Taraxacum officinale Wigg*) (roots in 49 and leaves in 15 species), *Urtica dioica* L. (leaves in 49 and roots in 1 species), *Rosa* spp. (fruits in 44 species), *Hypericum* spp. (aerial parts in 37 and flowers in 6 species), *Galega officinalis* L. (aerial parts in 41 species and seeds in one species), *Mentha × piperita* L. (leaves in 29 and aerial parts in 11 species), *Arctium* spp*.* (roots in 34 and leaves in 2 species), and *Fragaria vesca* L. (leaves in 26, aerial parts in 5, and roots in 1 species). Although the main plant parts used in species are the same as those recorded in the State Pharmacopoeia of the Russian Federation, multiple parts of some plants are utilized. Particularly, aerial parts, fruits, and roots (*Petroselinum crispum* (Mill.) Fuss); leaves, flowers, and roots (*Sambucus nigra* L.); and roots and leaves (*Cichorium intybus* L*., Rubus caesius* L., and *Arctium* spp.) have been used.

### The Popular Combinations of Medicinal Plants and Rationality for Combination

It is believed that, in medical species, several herbs work together harmoniously to achieve an ideal therapeutic effect. Modern studies on the mechanisms of activities of individual plant extracts support the rationality of empirically composed polyherbal mixtures in traditional medicine. Furthermore, we discuss the most frequent combinations of plants used in medical species in light of their mechanisms of action. The most frequently mentioned binary combinations of plants in medical species used for the treatment of diabetes are specified in Table 2.

**TABLE 2:** The most frequently mentioned binary combination of plants in medical species used for the treatment of diabetes.

	*Vaccinium myrtillus* L. leaves	*Phaseolus vulgaris* L. pericarp	*Urtica dioica* L. leaves	*Galega officinalis* L. aerial part	*Taraxacum campylodes* G.E.Haglund. roots	*Fragaria vesca* L*.* leaves	*Rosa* spp. fruits	*Arctium spp.* root	*Vaccinium vitis-idaea* L*.* leaves	*Hypericum spp.*	*Mentha × piperita* L*.* leaves
*Phaseolus vulgaris* L. pericarp	40										
*Urtica dioica* L. leaves	26	17									
*Galega officinalis* L. aerial part	22	17	14								
*Taraxacum campylodes* G.E.Haglund. roots	21	19	19	9							
*Fragaria vesca* L*.* leaves	14	8	8	4	5						
*Rosa* spp. fruits	16	10	6	4	4	10					
*Arctium spp.* roots	15	9	5	5	2	4	8				
*Vaccinium vitis-idaea* L*.* leaves	13	8	5	10	2	4	7	8			
*Hypericum* spp.	15	12	8	4	7	8	9	5	4		
*Mentha × piperita* L*.* leaves	12	6	2	4	4	3	10	5	4	7	
*Linum usitatissimum* L. seeds	11	11	4	2	5	5	9	5	5	5	3

The leading binary combination noted in medical species (Table 2) comprises the leaves of *Vaccinium myrtillus* L. and pericarp of *Phaseolus vulgaris* L. (quoted in 40 medical species). The leaves of *Vaccinium myrtillus* were widely used in Europe for the treatment of diabetes for a long time before the discovery of insulin (Helmstädter and Schuster, 2010). The extract was considered a potent inhibitor of α-glucosidase, with an IC50 value not statistically significantly different from the IC50 of acarbose (Bljajić et al., 2017), and to decrease blood glucose (Cignarella et al., 1996) and glycated hemoglobin (Sidorova et al., 2017). An extract from the pericarp of *Phaseolus vulgaris* L. significantly decreases the levels of plasma triacylglycerol and low-density lipoprotein in the blood (Pari and Venkateswaran, 2004; Sidorova et al., 2017), lowers blood glucose and cholesterol in the blood, and inhibits α-amylase activity (Micheli et al., 2019). The normalization of lipid profiles and systemic antioxidant effects are also attributed to this plant by other scientists (Venkateswaran et al., 2002; Helmstädter, 2010; Almuaigel et al., 2017).

The next most popular binary combination includes leaves of *Vaccinium myrtillus* L. and leaves of *Urtica dioica* L. (noted in 26 medical species). In addition to *Vaccinium myrtillus* L., the extract from the leaves of *Urtica dioica* L. reduces glycemia, potentiates the activity of insulin, enhances the utilization of glucose (El Haouari and Rosado, 2019), protects pancreatic β-cells (Golalipour and Khori, 2007), inhibits intestinal glucose absorption (Bnouham, et al., 2003), and shows total cholesterol-lowering activity (Avci et al., 2006). Eight weeks of treatment of patients with type 2 diabetes with *Urtica dioica* extract resulted in reductions in plasma glucose, triglycerides, and liver serum glutamic-pyruvic transaminase. Meanwhile, NO and superoxide markedly increased (Behzadi et al., 2016).

The combination of the leaves of *Vaccinium myrtillus* L. and aerial parts of *Galega officinalis* L. is described for 22 medical species. Complimentarily to *Vaccinium myrtillus* L., the extract from *Galega officinalis* L. reduces blood glucose, promotes the recovery of pancreatic β-cells (Sabeva et al., 2004; Shojaee et al., 2015), increases insulin-stimulated glucose uptake, activates peroxisome proliferator-activated receptor (PPARγ) (Christensen et al., 2009), normalizes neutrophils, reduces lymphoblast numbers, and inhibits the apoptosis of lymphocytes, which prevents the development and progression of diabetic complications (Nagalievska et al., 2018). *Galega officinalis* L. is a world-renowned herbal lineage containing metformin (Bailey, 2017). It should be noted that the efficacy of the binary combination of extracts of *Vaccinium myrtillus* L. and *Galega officinalis* L. was confirmed *in vivo*. A dry extract of this combination (50 mg/kg) was intragastrically administered to rats with streptozotocin (STZ)-induced diabetes. After 21 days of treatment, histological examination evidenced the recovery of degenerative and focal necrobiotic changes in the parenchymatous structures of the liver and kidneys and their blood flow caused by STZ (Kurylo et al., 2018). In another study, the same combination of extracts was administered intragastrically to rats with STZ-induced diabetes for 28 days. After 7 days of treatment, blood glucose was decreased by 69% compared with control, while after 28 days of treatment, blood glucose was decreased by 25% compared with control. A positive effect of the combination was also observed in the oral glucose tolerance test (OGTT) (Kurylo et al., 2020). The rationality of the *Vaccinium myrtillus* L. and *Galega officinalis* L. combination was confirmed in a number of experiments by Achilov (2020). A screening study of the individual extracts (*Vaccinium myrtillus* L. (50 mg/kg) and *Galega officinalis* L. (70 mg/kg)) and a combination at 50 mg/kg showed that, in OGTT in rats, the combination of the extracts decreased glucose more effectively than the individual extracts. The strongest effect was observed at 60 min. Studies on models of epinephrine-induced hyperglycemia in rats, alloxan-induced diabetes in rats, and dithizone-induced diabetes in rabbits showed hypoglycemic activity of the combined extract at 60 mg/kg (Achilov, 2020). Roots of *Taraxacum campylodes* G.E. Haglund. in combination with the leaves of *Vaccinium myrtillus* L. are contained in 21 medical species; the former in combination with the leaves of *Urtica dioica* L. are used in 19 medical species, and the same in combination with the pericarp of *Phaseolus vulgaris* L. are used in 19 medical species (Table 2). The leaves of *Taraxacum campylodes* G.E. Haglund. are also used in binary combinations with the leaves of *Vaccinium myrtillus* L. and leaves of *Urtica dioica* L. The *Taraxacum campylodes* G.E. Haglund. root extract inhibits adipogenesis, regulates lipid metabolism by inhibiting fat accumulation, increases lipolysis, and normalizes cholesterol and triglyceride levels (García-Carrasco et al., 2015). The leaf extract inhibited pancreatic lipase *in vitro* and *in vivo*, reduced triglyceride levels in the plasma of mice (Zhang et al., 2008), and stimulated the release of insulin in pancreatic β-cells (Hussain et al., 2004).

All the other binary combinations are used in fewer than 10% of the medical species discussed in this review. Therefore, we summarize the mechanisms of activities of the other plants cited in Table 2 separately in Table 3. The above-mentioned literature data suggest that binary combinations provide additive/synergistic effects.

**TABLE 3:** Complimentary mechanisms of most often used medicinal plants in binary combination in medical species used for the treatment of diabetes.

Medicinal plant and part used	Mechanisms	References
*Fragaria vesca* L*.* leaves	Decrease of total cholesterol, triglycerides, low- and high-density lipoproteins, normalization in antioxidant system (decrease of malondialdehyde and increase of superoxide dismutase); inhibition of α-glucosidase and α-amylase enzyme activity; reduce blood glucose level.	Tassa et al. (2012), Takács et al. (2020)
*Rosa* spp. fruits	Reduce blood glucose level, regulate lipid metabolism by inhibiting fat accumulation (mainly visceral), decrease serum triglycerides, regeneration of pancreas β-cells, increase expression of insulin-dependent genes Gck and Ptp1b.	Ninomiya et al. (2007), Orhan et al. (2009), Taghizadeh et al. (2016), Fattahi et al. (2017), Bahrami et al. (2020)
*Arctium* spp. *r*oots	Decrease of blood glucose, increase of insulin synthesis, suppression of lipid synthesis by activating 5′-adenosine monophosphate activated protein kinase, regulated the expression of sterol regulatory element-binding protein-1 and stearoyl-CoA desaturase.	Kuo et al. (2012), Ahangarpour et al. (2017), Chen et al. (2020)
*Vaccinium vitis-idaea* L*.* leaves	Decrease of blood glucose, increase of insulin synthesis, decrease of triglycerides, and high-density lipoproteins.	Barnaulov (2008), Zagayko et al. (2016)
*Hypericum* spp.	Reduce blood glucose level, inhibit pancreatic lipase, fat accumulation reduce hypercholesterolemia, lowered total cholesterol and low-density cholesterol, triglycerides, improved the insulin sensitivity, reduce expression of Dgat1, ColV, andLp1 genes involved in the biosynthesis of triglycerides	Arokiyaraj et al. (2011), Husain et al. (2011), Hernández-Saavedra et al. (2016), Tokgöz and Altan (2020)
*Mentha × piperita* L*.* leaves	Decrease of serum glucose, cholesterol, triglycerides, very low density lipoprotein, low density lipoprotein. Increase the high density lipoprotein cholesterol levels; inhibit glucosidase and tyrosinase	Barbalho et al. (2011), Bayani et al. (2017), Pavlić et al. (2021), Zeljković et al. (2021)
*Linum usitatissimum* L. seeds	Decrease blood glucose and polyphagia, control of lipid peroxidation (thiobarbituric acid-reactive substances) and antioxidant enzymes (glutathione peroxidase, superoxide dismutase, and catalase), inhibit glucosidase and α-amylase	Bhat et al. (2011), Bouzghaya et al. (2020)

Notably, the binary combinations of the leaves of *Vaccinium myrtillus* L. and pericarp of *Phaseolus vulgaris* L.; leaves of *Vaccinium myrtillus* L. and roots of *Arctium* spp*.*; and roots of *Taraxacum campylodes* G.E. Haglund. and leaves of *Mentha × piperita* L*.* occur as self-sufficient medical species (Table 1).

**FIGURE 2:**
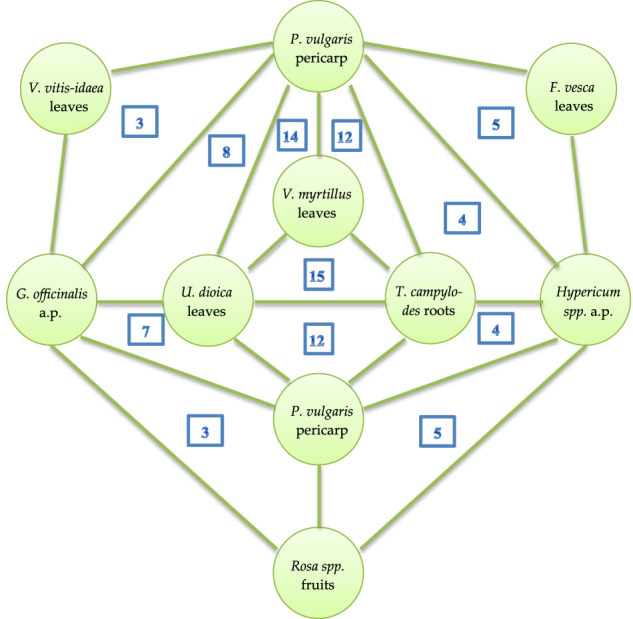
Leading ternary plant combinations in medical species. a.p.—aerial part; the numbers inside triangles indicate how often these ternary combinations occur in medical species.

Deeper analysis of all the medical species allowed us to identify leading ternary plant combinations, which are presented in Figure 2. It is not surprising that the leaves of *Vaccinium myrtillus* L., pericarp of *Phaseolus vulgaris* L., roots of *Taraxacum campylodes* G.E. Haglund., leaves of *Urtica dioica* L., and aerial parts of *Galega officinalis* L. are principal members of the ternary combinations.

### In Silico Probability of Antidiabetic Activity for Principal Compounds Identified in Selected Plants

The progress in computer science in symbiosis with modern pharmacology has led to the active implementation of computer-based prognosis for the activity of herb-derived compounds. Using an *in silico* approach, we analyzed the probability of antidiabetic activity for the principal compounds identified in the plants most often mentioned in binary and ternary combinations.

The prediction was performed using the free web resource PASS Online (Prediction of Activity Spectra for Substances). The prediction is based on an analysis of the structure and biological activity relationships for more than 300,000 organic compounds (Filimonov et al., 2014). Table 4 includes the prediction results for the antidiabetic efficacy of active compounds from selected medicinal plants with appropriate probability values: the likelihood of the given activity being revealed (Pa) or not revealed (Pi). If Pa>0.5, the substance is very likely to exhibit the activity (Lagunin et al., 2000).

**TABLE 4:** The active compounds from medicinal plants most often used in combinations in medical species and probability of predicted antidiabetic mechanisms assitiated with these compounds.

Medicinal plant/ (abbreviation)	Active compound	Mechanism of action	P_a_	P_i_
*Vaccinium myrtillus* L. (VM)	Isoorientin	Antidiabetic	0.806	0.005
	Vitexin-2"-rhamnosid	α-glucosidase inhibitor	0.854	0.001
		Antidiabetic	0.767	0.005
	Inositol	Sugar-phosphatase inhibitor	0.961	0.002
*Phaseolus vulgaris* L. (PV)	Isoorientin	Antidiabetic	0.806	0.005
	Myricetin	Lipid peroxidase inhibitor	0.836	0.003
		β-glucuronidase inhibitor	0.679	0.005
*Eleutherococcus senticosus* (Rupr. & Maxim.) Maxim. (ES)	Syringin	Antidiabetic	0.684	0.007
		Hypolipemic	0.674	0.016
	Eleutheroside E	Sugar-phosphatase inhibitor	0.887	0.005
*Hypericum perforatum* L. (HP)	Hyperoside	Antidiabetic	0.661	0.008
		Lipid peroxidase inhibitor	0.976	0.002
		Sugar-phosphatase inhibitor	0.874	0.006
		α-glucosidase inhibitor	0.842	0.001
*Aralia elata* (Miq.) Seem (AE)	Araloside A	Antidiabetic	0.639	0.009
		Hypolipemic	0.955	0.003
		Insulin promoter	0.753	0.004
	Araloside B	Lipid peroxidase inhibitor	0.969	0.002
		Hypolipemic	0.953	0.003
		α-glucosidase inhibitor	0.932	0
	Araloside C	Hypolipemic	0.952	0.003
*Fragaria vesca* L. (FV)	Taxifolin-3-O-arabinofuranoside	Antidiabetic	0.617	0.011
		Lipid peroxidase inhibitor	0.95	0.002
		Antihypercholesterolemic	0.901	0.003
*Urtica dioica* L. (UD)	2-O-caffeoylmalic acid	Lipid metabolism regulator	0.836	0.005
	Quercetin p-coumaroyl glucoside	Lipid peroxidase inhibitor	0.978	0.002
		α-glucosidase inhibitor	0.729	0.001
*Galega officinalis* L. (GO)	Galegine	Sugar-phosphatase inhibitor	0.632	0.047
		Glucose oxidase inhibitor	0.691	0.025
	Phytol	Lipid metabolism regulator	0.828	0.005
		Hypolipemic	0.68	0.015
*Taraxacum campylodes* G.E.Haglund. (TC)	Taraxacin	β-glucuronidase inhibitor	0.619	0.011
*Rosa* spp. (Rsp)	Lycopene	Sugar-phosphatase inhibitor	0.794	0.017
		Lipid metabolism regulator	0.880	0.004
*Arctium* spp*.* (Asp)	Arctigenic acid	Insulin promoter	0.579	0.017
*Vaccinium vitis-idaea* L. (VVI)	Hydroquinone	Sugar-phosphatase inhibitor	0.906	0.004
		Inulinase inhibitor	0.690	0.004
*Mentha × piperita* L. (MP)	Menthol	Insulin promoter	0.773	0.004
		Sugar-phosphatase inhibitor	0.804	0.016
*Linum usitatissimum* L. (LU)	Gallic acid	Sugar-phosphatase inhibitor	0.941	0.003
		Glucan endo-1.6-beta-glucosidase inhibitor	0.933	0.002

**FIGURE 3:**
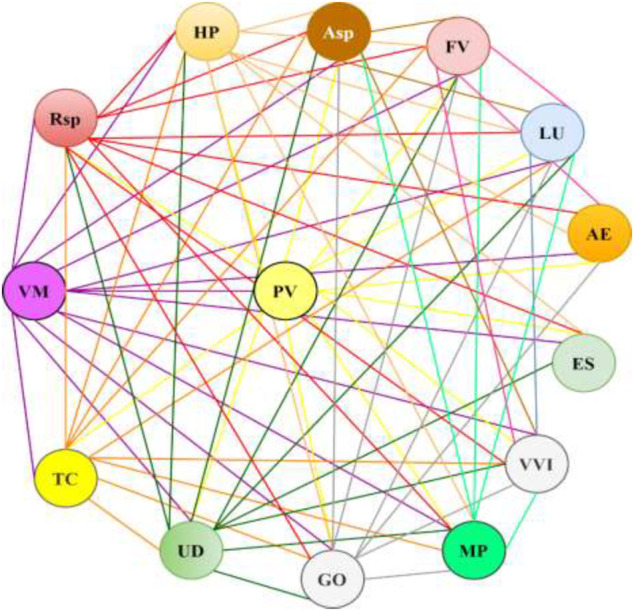
The combinations of the medicinal plants with a high probability of antidiabetic effects in medical species. The plant abbreviations are presented in Table 4.

The predicted Pa values for the active compounds identified in the most frequently used combinations of plants in medical species were over 0.5 and ranged from 0.619 for compounds from *Fragaria vesca* L. up to 0.976 for compounds from *Hypericum perforatum* L. (Table 4). The highest Pa values were found for compounds derived from *Urtica dioica* L., *Hypericum perforatum* L., *Vaccinium myrtillus* L., *Fragaria vesca* L., *Linum usitatissimum* L., and *Vaccinium vitis-idaea* L. (Pa>0.9). The diagram in Figure 3 demonstrates crosslinks between medicinal plants with a high probability of predicted antidiabetic effects (Table 4) used in medical species.

The calculated data support the rationality of the traditional use of medical species for the treatment of diabetes and its complications. Nevertheless, the chemical principles responsible for the observed effects are rarely studied. Except for the success story of metformin derived from *Galega officinalis* L., no other compounds are on the market. Systematic studies of the combinatory action of different plant decoctions/infusions, as well as plant-derived compounds, are needed.

### Specificity of Medical Species Used in Russia

Several plants used in Russian traditional medicines for the treatment of diabetes and its complications are widely known in other countries. The leaves of *Urtica dioica* L., pericarp of *Phaseolus vulgaris* L., leaves of *Vaccinium myrtillus* L., and leaves and roots of *Taraxacum campylodes* G.E. Haglund. are among the most frequently used components for the management of diabetes by herbalists in Croatia (Končić and Bljajić, 2019). *Phaseolus vulgaris* is a well-known antidiabetic plant in the Ayurveda and Unani medicine systems (Ganesan and Xu, 2017). It is widely used in medicine in Poland (Łabuda et al., 2017). The antidiabetic potential of *Urtica dioica* is well documented in Arabic traditional medicine (Said et al., 2008). *Galega officinalis* L. has been used for the treatment of diabetes in Bulgaria (Petkov, 1982), Italy (Leporatti and Ivancheva, 2003), and Iran (Nowbandegani et al., 2015).

Unlike in other systems of medicine, the juices of some plants (the berries of *Viburnum opulus* L., *Solanum tuberosum* L., and *Daucus sativus* Roehl. and birch sap) have been used for preparing some medical species. Interestingly, eleven medical species contain seaweeds (*Cystoseira barbata* (Stackh.) C. Agardh; *Saccharina latissima* (L.) C.E.Lane, C.Mayes, Druehl & G.W.Saunders; *Fucus vesiculosus* L.), and one species contains lichen (*Cetraria islandica* L.). It is noteworthy that several adaptogenic plants have been used in medical species. Besides the common properties of promoting the adaptability, resilience, and survival of living organisms under stress (Panossian et al., 2021), each adaptogen has some specific activity. In particular, *Oplopanax elatus* (Nakai) Nakai lowered blood glucose and increased insulin levels *in vivo* (Molokovskii et al., 2002). Glucose- and cholesterol-lowering effects, decreased glycosuria, and increased insulin levels were observed in diabetic patients after complex therapy with *Oplopanax elatus* (Nakai) Nakai (Klimakova and Kazmanm, 1962). *Aralia elata* (Miq.) Seem decreases blood glucose, inhibits insulin resistance, alleviates hyperlipidemia *in vivo* (Hwang et al., 2015), and improves blood glucose and lipid metabolism in humans (Abidov et al., 2006). The activity could be associated with aralosides (Pa, 0.639–0.969, Table 4). *Eleutherococcus senticosus* (Rupr. et Maxim.) Maxim. and its active compounds lowered blood glucose, increased glycogen levels, ameliorated insulin resistance, and increased insulin levels *in vivo* (Molokovskii et al., 2002; Niu et al., 2008; Ahn et al., 2013). The activity is associated with syringing and eleutheroside E (Pa, 0.684 and 0.887; Table 4). These adaptogens are not only used in medical species in traditional medicine but are included in the officinal medical species “Arfazetin” (Table 1).

### Principles for Compilation of Medical Species

Due to the specific location of Russia, Russian herbal medicine has adopted the philosophy of Eastern traditional medicine and the pragmatic approach of Western medicine. One of the main principles for the compilation of the formulas used in traditional Chinese medicine is described in Shen-nong Ben-Cao Jing. An effective formula should be based on a strong monarch, accompanied by a minister, assistant, and guide, which mimics a well-organized society (Xin et al., 2014; Xutian et al., 2014). However, this principle is difficult to follow in practice, due to the multiple symptoms of diseases and polyfunctionality of medicinal plants. Therefore, many formulas of TCM contain secrets that are not always explained by rationality (Wang et al., 2021).

The philosophy and conceptualization for the compilation of medical species in Russian medicine are not well described. After a comprehensive medical examination of a patient, a Russian phytotherapeutic doctor initially prescribes a basic medical species, which includes the plants that lower blood glucose. The binary and triple combinations emphasized in this review can be regarded as basic mixtures. Diabetes is often accompanied by obesity. Therefore, the basic mixture is fortified with plants reducing hypercholesterolemia. In the case of hypertension, the species include anti-hypertensive plants. The practical doctors also take into account the peculiarities of the gastrointestinal tracts of the patients. In this respect, medical species can include plants with astringent or laxative properties. To prevent allergic reactions, doctors recommend taking a basic mixture for a week and then continuously increasing the number of plants in the medical species one by one. Plants with antiallergic properties are sometimes included in the mixtures (Kovaleva, 1972; Ladynina and Morozova, 1987; Ladynina and Morozova, 1990).

This approach could be illustrated by the following medical species frequently cited in the literature. The species 4.39 (Table 1) includes a synergistic combination of *Vaccinium myrtillus* L. and *Galega officinalis* L., which effectively decreases glucose levels (Achilov, 2020; Kurylo et al., 2020). These basic plants have antidiabetic properties. *Frangula alnus* Mill. acts as a laxative, and *Betula pendula* Roth. acts as a diuretic (Belodubrovskaya et al., 2004). Another species, 4.40 (Table 1), besides the synergistic combination of *Vaccinium myrtillus* L. and *Galega officinalis* L., includes *Phaseolus vulgaris* L., which reduces plasma triacylglycerol and low-density lipoprotein, and lowers blood glucose and cholesterol (Pari and Venkateswaran, 2004; Sidorova et al., 2017; Micheli et al., 2019). These basic plants ensure the antidiabetic effect, while *Mentha × piperita* L. additionally provides anti-hypertensive, antiallergic, and spasmolytic effects (Mahendran and Rahman, 2020). The species 5.10 (Table 1) comprises 5 plants. The power of the basic mixture of the synergistic combination of *Vaccinium myrtillus* L. and *Galega officinalis* L. and *Phaseolus vulgaris* L. is reinforced by *Taraxacum campylodes* G.E. Haglund., which inhibits adipogenesis and fat accumulation (García-Carrasco et al., 2015). Additionally, *Urtica dioica* L. potentiates the activity of insulin and enhances the utilization of glucose (El Haouari and Rosado, 2019). We understand that the interpretation of the rationality of the above-mentioned medical species compilation took into account the results of modern research. Apparently, the architects of the discussed medical species compiled them according to their own experience and knowledge. Similar to the TCM formulas, some Russian medical species also have secrets that are yet to be deciphered. Nevertheless, the knowledge and practical experience of Russian traditional medicine were successfully utilized for the development of medical species used in officinal medicine.

### Medical Species Used in Russian Officinal Medicine

In Russia, medical species are part of officinal medicine. Although medical species are available as OTC products, consultations with phytotherapeutic doctors are helpful and will lead to more effective results. Among the 227 medical species discussed in this review, only two, “Arfazetin” and “Myrphasinum”, are approved for use in officinal medicine. Both species are recommended in the mild form of diabetes. The medical species “Arfazetin” was developed in the All-Union Institute of Medicinal and Aromatic Plants and was approved for medicinal use in 1986 (Ferubko et al., 2016). “Arfazetin” comprises seven medicinal plants (Table 1). In 1992, the composition of species was revised. The roots of *Aralia elata* (Miq.) Seem (syn. *Aralia mandshurica* Rupr. et Maxim.) or roots and rhizomes of *Oplopanax elatus* (Nakai) Nakai were excluded. Instead of these plants, the roots and rhizomes of *Eleutherococcus senticosus* (Rupr. et Maxim.) Maxim. were included in species at the same rate. A new species was named “Arfazetin-E”. Both these species (“Arfazetin” and “Arfazetin-E”) are now included in the State register (2021).

“Myrphasinum” was developed in 1985 by scientists from the first Moscow medical institute, named after I.M. Sechenov (Fas'kov et al., 1991), and was approved for medicinal use in 1991. The composition is complicated and includes the 12 medicinal plants (Table 1).

According to the regulatory requirements of the USSR/Russia, medical species are subject to preclinical and clinical evaluations of safety and efficacy.

### Preclinical and Clinical Data

The efficacy of “Arfazetin” was studied in several experiments *in vivo*. Rats with alloxan-induced diabetes were administered an infusion of “Arfazetin” (10 ml/kg per day, orally) five days before alloxan injection and seven days after injection. Distilled water was administered in the control group. The blood glucose level, elevated by alloxan, was decreased in “the Arfazetin”-treated rats by 24 and 38% when compared with control on the third and seventh days, respectively, after alloxan injection. The total cholesterol, creatinine, and malondialdehyde in the blood and liver were decreased by 27, 37, 30, and 30%, respectively, compared with the control group on the seventh day after treatment. The treatment of rats with “Arfazetin” led to an increase in serum immunoreactive insulin and C-peptide after glucose load by 22 and 55%, respectively, when compared with the control group (Azhunova et al., 2001). Similar results were observed in a prolonged study. The oral administration of “Arfazetin” (5 ml/kg of infusion) to rats with alloxan-induced diabetes over 30 days (7 days before alloxan induction and 23 days after injection) resulted in decreases in blood glucose of 46 and 39%, respectively, compared with the control group on the 15th and 30th days of the experiment. On Day 30, glycogen in the liver was increased by 17% when compared with the control group (Ishankulova et al., 2013). A further study by the same group evidenced the lipid-lowering properties of “Arfazetin” (infusion, 5 ml/kg, orally). The total cholesterol in the blood decreased by 33% when compared with the negative control after 30 days of the treatment of rats with alloxan-induced diabetes. The levels of triglycerides and low- and high-density lipoproteins normalized and were equal to those in the intact group after 30 days of treatment with the infusion of “Arfazetin” (Ishankulova and Yuldasheva, 2019). In another study, the effects of “Arfazetin” on energy metabolism in rats were reported. Rats with alloxan-induced diabetes were treated with the infusion of “Arfazetin” (10 ml/kg, orally, daily) for 21 days. The control group received the same volume of distilled water. The treatment of rats with "Arfazetin" resulted in a double increase in adenosine triphosphate production in the liver when compared with control, starting from the seventh day of the experiment. The concentration of lactic acid decreased by 1.7 fold, while the activity of pyruvate kinase increased by 1.5 fold when compared with the control group after 21 days of treatment. The authors suggest positive effects of “Arfazetin” on energy metabolism (Lemza et al., 2014). The blood glucose in rats with alloxan-induced diabetes was decreased by 3.2 fold when compared with control at 3 h after the intragastric administration of a dry extract of the medical species “Arfazetin” (1,200 ng/kg). The efficacy of the extract was equal to that of gliclazide (50 mg/kg, intragastric administration) (Kvasova, 2011).

The acute and chronic toxicity of “Arfazetin” was studied in mice after oral administration. The LD_50_ for the dry extract of “Arfazetin” was 24 g/kg (acute toxicity). No signs of toxicity were observed in the mice after 30 days of the administration of the “Arfazetin” infusion and dry extract at 1,200 mg/kg (there times a day every 4 h) (Kvasova et al., 2010).

The antidiabetic potential of the medical species “Myrphasinum” was investigated in rats. Diabetes was modulated by the subcutaneous injection of alloxan. The glucose level in the blood was increased from 5.6 mmol/L (intact group) to 9.55 mmol/L, body weights were decreased, and the rats had no appetite. The aqueous infusion of “Myrphasinum” (25 g/L) was administered to rats by an intragastric route at the dose of 5 ml/kg three times per day for two weeks. The control group was administered saline. The blood glucose in the treated group was decreased to 3.4 mol/L (vs. an increase up to 10.7 ml/L in the control group) two weeks after the beginning of treatment. The body weights and appetite were improved. Meanwhile, 25% of the animals in the control group died. The animals were observed for five extra weeks after the end of treatment with “Myrphasinum”. Three weeks after the end of treatment, the blood glucose in the treated group was equal to that in the intact group (5.77 mmol/L) and was stable until the fifth week (Grinkevich et al., 1997). In another study, outbred rats with alloxan-induced diabetes were orally administered 10 ml/kg of an infusion of “Myrphasinum” 3 times a day for three weeks. The control group received the same dose of normal saline. The treatment with “Myrphasinum” resulted in a statistically significant decrease in blood glucose by 26% compared with control. Glycogen in the liver and skeletal muscles was increased by 35 and 21% respectively, when compared with control and was equal to the level in an intact group (Dzhafarova, 2013). Subsequently, the efficacy of “Myrphasinum” in outbred rats with alloxan-induced diabetes was evaluated. The rats were treated orally with 10 ml/kg of an infusion of “"Myrphasinum” 3 times a day. The control group received normal saline. Metformin (5 mg/kg, 2 times a day) served as a positive control. The administration of “Myrphasinum” for 21 days did not affect the body weights of the rats, and no signs of toxicity were observed. The level of glucose in the “Myrphasinum”-treated group was decreased by 75% compared with control (vs. 59% in the metformin group). The insulin and C-peptide levels were dramatically reduced by 3 and 3.3 fold, respectively, in rats after alloxan injection in those treated with "Myrphasinum" (vs. 1.5 and 1.7 fold increases, respectively, in the animals treated with metformin) (Jafarova and Garayev, 2013).

We have found, in the available literature, only a few publications about clinical trials with medical species. The efficacy of “Arfazetin” was studied in a group of 32 patients (18–65 years old) with types I and II diabetes mellitus. “Arfazetin” was prepared in the form of an aqueous infusion (10 g in 400 ml) and administered in warm form at the dose of 1/3 glass, three times a day, 30 min before meals, for one month. The patients with type I diabetes (12 persons) were administered “Arfazetin” in combination with an appropriate dose of insulin and diet. The five patients with a mild form of type II diabetes were administered “Arfazetin” in combination with an appropriate diet. The group of 15 patients with a moderate form of type II diabetes were administered “Arfazetin” in combination with an appropriate dose of hypoglycemic drugs and diet. In the patients with type I diabetes treated with “Arfazetin”, a statistically significant decrease in blood glucose (by 38%) was registered at 11.00 pm when compared with 9.00 am of the same day. The effect was not cumulative. More pronounced results were observed in patients with type II diabetes. “Arfazetin” effectively ameliorated hyperglycemia. The doses of hypoglycemic drugs were reduced in 7 patients. In two patients, it was possible to maintain normal blood glucose levels without hypoglycemic drugs (Korotkova et al., 1988)**.**

A “Myrphasinum” infusion was used in clinical praxis for the therapy of patients with and without diabetes decompensation. The treatment of patients with diabetes in the compensation stage resulted in statistically significant decreases in glucose by 15 and 44% in the blood and urine, respectively. Cholesterol and B-lipoproteins were decreased by 18 and 21%, respectively. The effects of “Myrphasinum” in patients with diabetes in the decompensation stage were less pronounced (Fas'kov et al., 1991). However, the data provided in the inventor’s certificate are limited and lacking other details.

The comparative efficacy of “Arfazetin” and “Myrphasinum” was studied in 57 patients with diabetes (22–76 years old) in an open clinical trial. The first group (26 persons) was treated with “Arfazetin”, while the second group (31 persons) received “Myrphasinum”. Basic therapy includes oral hypoglycemic drugs. “Myrphasinum” was considered more effective and resulted in a statistically significant decrease in blood glucose, surpassing “Arfazetin” in efficacy (Firsova et al., 1990). However, no more details were provided in this conference paper.

## Conclusion

In this review, we analyze the compositions and potential of medical species used in Russian traditional and officinal medicine for the treatment of diabetes and related diseases. Several species besides medicinal plants contain fresh juices from berries, birch sap, and seaweeds. Another aspect of medical species is the presence of adaptogens. The philosophy and conceptualization for the compilation of medical species in Russian medicine are not well described. We have highlighted the most common binary and triple combinations of plants exploited in medical species. These combinations can be considered base mixes. Other plants are added to the mixtures to improve the efficacy, treat associated disorders, improve gastrointestinal function, prevent allergic reactions, etc. Obviously, Russian phytotherapeutic doctors compile polyherbal mixtures according to their own experience and knowledge. Modern studies of the mechanisms of action and predicted activities of the principal compounds from medicinal plants support the rationality of polyherbal mixtures. However, the mechanisms are not well studied and reported due to the limited number of compounds. Deeper investigations including gene expression will enable a better understanding of molecular mechanisms and targets. Although a few studies have evidenced possible additive/synergistic effects of herbal mixtures, additional investigations with calculations of synergistic or additive indices will assist in providing a scientific foundation for the wider use of medical species for the therapy of diabetes. Even though most medical species comprise mixtures of three to six plants, other species also deserve careful study. It appears to us that the species with seven or more plants have rationality that is difficult to explain and some secrets that are yet to be deciphered. On the other hand, modern good praxis rules require the identification of all the plants in medical species. An increase in plants in the mixture requires advanced techniques for quality control. Notably, two medical species approved for use in officinal medicine include 7 and 12 plants. The efficacy of these species was investigated *in vivo*. However, all the activities were proved using only one model of alloxan-induced diabetes. Clinical trials were completed in small groups, and several details are not indicated in the reports. According to modern regulatory rules, additional pharmacological experiments and clinical trials are required for more detailed investigations of the mechanisms of action and the confirmation of efficacy. We believe that the scientifically based utilization of rich plant resources and knowledge of Russian herbal medicine can significantly contribute to the local economy as well as to the sectors seeking natural healing products.
